# Clinical significance and potential regulatory mechanism of overexpression of pituitary tumor-transforming gene transcription factor in bladder cancer

**DOI:** 10.1186/s12885-022-09810-y

**Published:** 2022-06-29

**Authors:** Jian-Di Li, Abdirahman Ahmed Farah, Zhi-Guang Huang, Gao-Qiang Zhai, Rui-Gong Wang, Jia-Lin Liu, Qin-Jie Wang, Guan-Lan Zhang, Zi-Long Lei, Yi-Wu Dang, Sheng-Hua Li

**Affiliations:** 1 Department of Pathology, The First Affiliated Hospital of Guangxi Medical University, No.6 Shuangyong Rd, Guangxi Zhuang Autonomous Region, 530021 Nanning, People’s Republic of China; 2 Department of Urology, The First Affiliated Hospital of Guangxi Medical University, No.6 Shuangyong Rd, Guangxi Zhuang Autonomous Region, 530021 Nanning, People’s Republic of China

**Keywords:** BLCA, PTTG1, Tumor microenvironment, Transcriptional regulation

## Abstract

**Background:**

Pituitary tumor transforming gene-1 (PTTG1) transcription factor is identified as carcinogenic and associated with tumor invasiveness, but its role in bladder cancer (BLCA) remains obscure. This research is intended to analyze the aberrant expression and clinical significance of PTTG1 in BLCA, explore the relationship between PTTG1 and tumor microenvironment characteristics and predict its potential transcriptional activity in BLCA tissue.

**Methods:**

We compared the expression discrepancy of PTTG1 mRNA in BLCA and normal bladder tissue, using the BLCA transcriptomic datasets from GEO, ArrayExpress, TCGA, and GTEx. In-house immunohistochemical staining was implemented to determine the PTTG1 protein intensity. The prognostic value of PTTG1 was evaluated using the Kaplan-Meier Plotter. CRISPR screen data was utilized to estimate the effect PTTG1 interference has on BLCA cell lines. We predicted the abundance of the immune cells in the BLCA tumor microenvironment using the microenvironment cell populations-counter and ESTIMATE algorithms. Single-cell RNA sequencing data was applied to identify the major cell types in BLCA, and the dynamics of BLCA progression were revealed using pseudotime analysis. PTTG1 target genes were predicted by CistromeDB.

**Results:**

The elevated expression level of PTTG1 was confirmed in 1037 BLCA samples compared with 127 non-BLCA samples, with a standardized mean difference value of 1.04. Higher PTTG1 expression status exhibited a poorer BLCA prognosis. Moreover, the PTTG1 Chronos genetic effect scores were negative, indicating that PTTG1 silence may inhibit the proliferation and survival of BLCA cells. With PTTG1 mRNA expression level increasing, higher natural killer, cytotoxic lymphocyte, and monocyte lineage cell infiltration levels were observed. A total of four candidate targets containing *CHEK2*, *OCIAD2*, *UBE2L3*, and *ZNF367* were determined ultimately.

**Conclusions:**

PTTG1 mRNA over-expression may become a potential biomarker for BLCA prognosis. Additionally, PTTG1 may correlate with the BLCA tumor microenvironment and exert transcriptional activity by targeting *CHEK2*, *OCIAD2*, *UBE2L3*, and *ZNF367* in BLCA tissue.

**Supplementary Information:**

The online version contains supplementary material available at 10.1186/s12885-022-09810-y.

## Background

Bladder cancer (BLCA) is the world’s ninth highest cause of cancer-related mortality among men and the second most prevalent malignancy of the human urinary tract in 2020 [1]. According to the cancer statistics 2022, there will be approximately 91,893 new cases of BLCA in China, with 42,973 cancer-related mortality [2]. The majority of BLCA patients are non-muscle-invasive [3]. Because these two forms of BLCA have fundamentally different biological features, there are major differences in disease onset, overall survival status, and therapeutic regimens [4]. Radical cystectomy is the primary therapeutic strategy for BLCA patients; however, it has a high postoperative recurrence rate, a high incidence of distant metastases, and a low 5-year survival rate [5]. Therefore, it is urgent to develop novel biomarkers that may be used in early diagnosis, prognostic evaluation, and therapy to improve the survival outcomes of BLCA patients.

The pituitary tumor-transforming gene (PTTG) transcriptional factor (TF) is an oncogenic gene first isolated and discovered in rat cell lines [6]. According to the sequence in which they were identified, there are three PTTG isoforms, namely PTTG1, PTTG2, and PTTG3 [7]. PTTG1 is a multifunctional protein implicated and overexpressed in various endocrine-related malignancies, namely pituitary [8], uterine [9], breast [10], and ovarian tumors [11]. Previous research has linked it to the passage of numerous cancer cell types through the metaphase-anaphase transition in the cell cycle process [12, 13]. In addition, PTTG1 is substantially connected with tumor invasiveness and is known to be a crucial gene associated with tumor metastasis, whose expression levels in normal human tissue are low [14]. Moreover, PTTG1 could promote the proliferation and metastasis potential of several human tumor types, such as colon cancer [15], esophageal cancer [16], and lung cancer [17], which suggests the pro-tumor role of PTTG1. However, the clinical significance and potential transcriptional regulatory mechanisms of PTTG1 in BLCA are still unclear and require further investigation.

Therefore, the goals of this study were to evaluate the overall expression level of PTTG1 in BLCA tissues and to investigate its potential clinical value in BLCA patients, as well as the relationship between PTTG1 and tumor immune infiltration, and its transcriptional activity in BLCA tissues.

## Methods

### BLCA tissue samples

Surgery-dissected BLCA and normal bladder tissue specimens were collected from the First Affiliated Hospital of Guangxi Medical University. The inclusion criteria were as follows, (I) the pathological type of BLCA tissue samples should be transitional cell carcinoma; (II) sufficient tissue samples should be prepared for performing tissue microarray and immunohistochemical staining. This study had been approved by the Ethics Committee of the First Affiliated Hospital of Guangxi Medical University (2022-KT-GUOJI-146).

### In-house immunohistochemistry

To explore the protein expression status of PTTG1 in BLCA, immunohistochemical staining was conducted by using the in-house BLCA and non-BLCA tissue samples. The rabbit polyclonal antibody to PTTG1 (#orb374037) was purchased from Biorybt Co., Ltd. (https://www.biorbyt.com/).

### The human protein atlas (THPA)

The protein expression of PTTG1 in BLCA tissues and normal bladder tissues was also inquired by using THPA (https://www.proteinatlas.org/). A total of two kinds of antibodies were selected, including HPA045034 and CAB008373.

### Cancer dependency map (DepMap)

The DepMap portal (https://depmap.org/portal/) enables researchers to have a broad view of genetic and pharmacologic dependencies in cancers [18]. DepMap provides large-scale clustered regularly interspaced short palindromic repeats (CRISPR) screen resources, which helps in depicting the roadmap of oncology therapeutic targets [19]. Herein, DepMap was used to validate the expression of PTTG1 mRNA in a total of 36 BLCA cell lines. Moreover, PTTG1 CRISPR knockout and RNA interference (RNAi) knockout data were downloaded from DepMap to explore the perturbation effects that PTTG1 has on BLCA cell lines.

### Public transcriptome database

Global BLCA gene microarrays and mRNA sequencing data sets were downloaded from the Gene Expression Omnibus (GEO), ArrayExpress, The Cancer Genome Atlas (TCGA), and The Genotype-Tissue Expression project. The inclusion standards were set as follows, (I) the specimen should be human primary BLCA tissue; (II) each platform data set should contain no less than three BLCA samples and three non-BLCA samples. The exclusion standards were as follows, (I) the probe ID annotation information was missing; (II) the patient had received preoperative treatment. The enrolled data sets were assigned into different groups according to the affiliated platform. The data sets in each platform were integrated into a larger matrix, named platform matrix. The generated batch effect was removed by using Limma-voom and sva packages.

### Differential expression analysis

To portray the global BLCA differentially expressed genes (DEGs) map, the included platform matrices were utilized for calculating standardized mean difference (SMD). Up-regulated gene and down-regulated gene were defined as follows, (I) up-regulated gene: SMD > 0, *P* < 0.05; (II) down-regulated gene: SMD < 0, *P* < 0.05.

### Prognostic analysis

TCGA-BLCA patients were assigned to the PTTG1 high expression group and low expression group according to the optimal cutoff value. Overall survival (OS) and disease-free survival (DFS) Kaplan-Meier survival analysis were conducted to appraise the prognostic value of PTTG1 in BLCA patients by using the Kaplan-Meier Plotter (https://kmplot.com/analysis/index.php?p=service&cancer=pancancer_rnaseq).

### Microenvironment cell populations-counter (MCP-counter)

The tumor microenvironment (TME) takes an important part in the pathogenesis of malignant tumors [20]. The MCP-counter algorithm helps in the cell infiltration quantification of eight immune cells and two stromal cells [21]. The author first downloaded the level three TCGA-BLCA fragments per kilobase of transcript per million fragments mapped (FPKM) data, which were transformed into transcripts per million (TPM) data subsequently. The immune infiltration levels of immune and stromal cells of TCGA-BLCA patients were quantified. Immune, stromal, estimate, and tumor purity scores were calculated by using a method called Estimation of STromal and Immune cells in MAlignant Tumours using Expression data (ESTIMATE) [22]. Finally, the correlations between PTTG1, as well as its targeting genes, and immune cells were validated by using the TIMER (https://cistrome.shinyapps.io/timer/) analysis tool.

### Single-cell RNA sequencing (scRNA-seq) dataset analysis

BLCA scRNA-seq dataset GSE135337 was downloaded from GEO [23]. GSE135337 included the single cells mRNA profiles of seven primary BLCA male patients (10X Genomics platform). A total of seven mRNA profiles were aggregated, filtered, and normalized. The filtered criteria were feature RNA > 50 and mitochondria percentage < 5. After principal component (PC) analysis, a total of 16 PC were selected for performing t-distributed Stochastic Neighbor Embedding (tSNE) nonlinear dimensionality reduction analysis. The marker genes of each clustered cell population were identified and were annotated by using the SingleR package. Subsequently, epithelial cells were selected for pseudotime analysis by using monocle, and the cell fate-related genes were identified and functionally annotated by Gene Ontology.

### Chromatin immunoprecipitation followed by sequencing (ChIP-seq) data analysis

The putative transcriptional targets of PTTG1 were downloaded from CistromeDB (http://cistrome.org/db/#/) (ID: 63264 and 63265) [24, 25]. The transcriptional targets of PTTG1 were acquired by intersecting cell fate-related genes, BLCA DEGs, and putative TF targets. The interaction network between PTTG1 TF and the intersected targets was analyzed by using STRING v11.5 (https://string-db.org/) [26]. The most closely interacted genes were predicted to be PTTG1 transcriptional targets.

### Functional enrichment analysis

The author performed functional enrichment analysis to explore the potential roles of PTTG1 in cancer phenotypes by interacting with different targets. TCGA-BLCA patients were assigned to the high expression group or the low expression group according to the expression value of PTTG1 targets. Limma package was used to conduct differential expression analysis. The identified DEGs between such groups, which were regarded as PTTG1-target-related genes, were functionally enriched by Gene Ontology and Kyoto Encyclopedia of Genes and Genomes.

### DNA methylation analysis

The DNA methylation levels of PTTG1, as well as its transcriptional targets, were analyzed by using MethSurv (https://biit.cs.ut.ee/methsurv/) [27–29]. Kaplan-Meier survival analysis was performed to appraise the prognostic value of PTTG1 methylation. Moreover, the correlation between PTTG1 mRNA expression level and PTTG1 methylation level was investigated by using cBioPortal (https://www.cbioportal.org/).

### Connectivity map (CMap)

To identify several potential small molecules for treating BLCA cells, drug repurposing analysis was performed by using the Connectivity Map (CMap) analysis tool [30]. Using the up-regulated transcriptional targets of PTTG1 as gene set input, the prospective therapeutic molecules for treating BLCA cells were identified by calculating the connectivity scores. A negative score indicates that the aberrant overexpression of PTTG1 targets may be counteracted by the identified small molecules.

### Statistical analysis

All the statistical analyses were completed by using R v4.0.4. Wilcoxon or Kruskal-Wallis tests were selected to compare the difference in PTTG1 expression levels between two or more than three groups. According to the result of the heterogeneity test, a fixed or randomized effect model was selected for pooling SMD values (a fixed-effect model for *I*^*2*^ ≤ 50%, while a randomized effect model for *I*^*2*^ > 50%). The discriminatory ability of PTTG1 was appraised by integrating true positive, false positive, false negative, and true negative rates into a summary receiver operating characteristic curve. The area under the curve (AUC) was used to indicate the discriminatory ability of PTTG1, where AUC < 0.7, 0.7 ≤ AUC < 0.9, and AUC ≥ 0.9 represented a weak, moderate, and strong ability.

## Results

### PTTG1 over-expression in BLCA tissue and cells

**Fig. 1:**
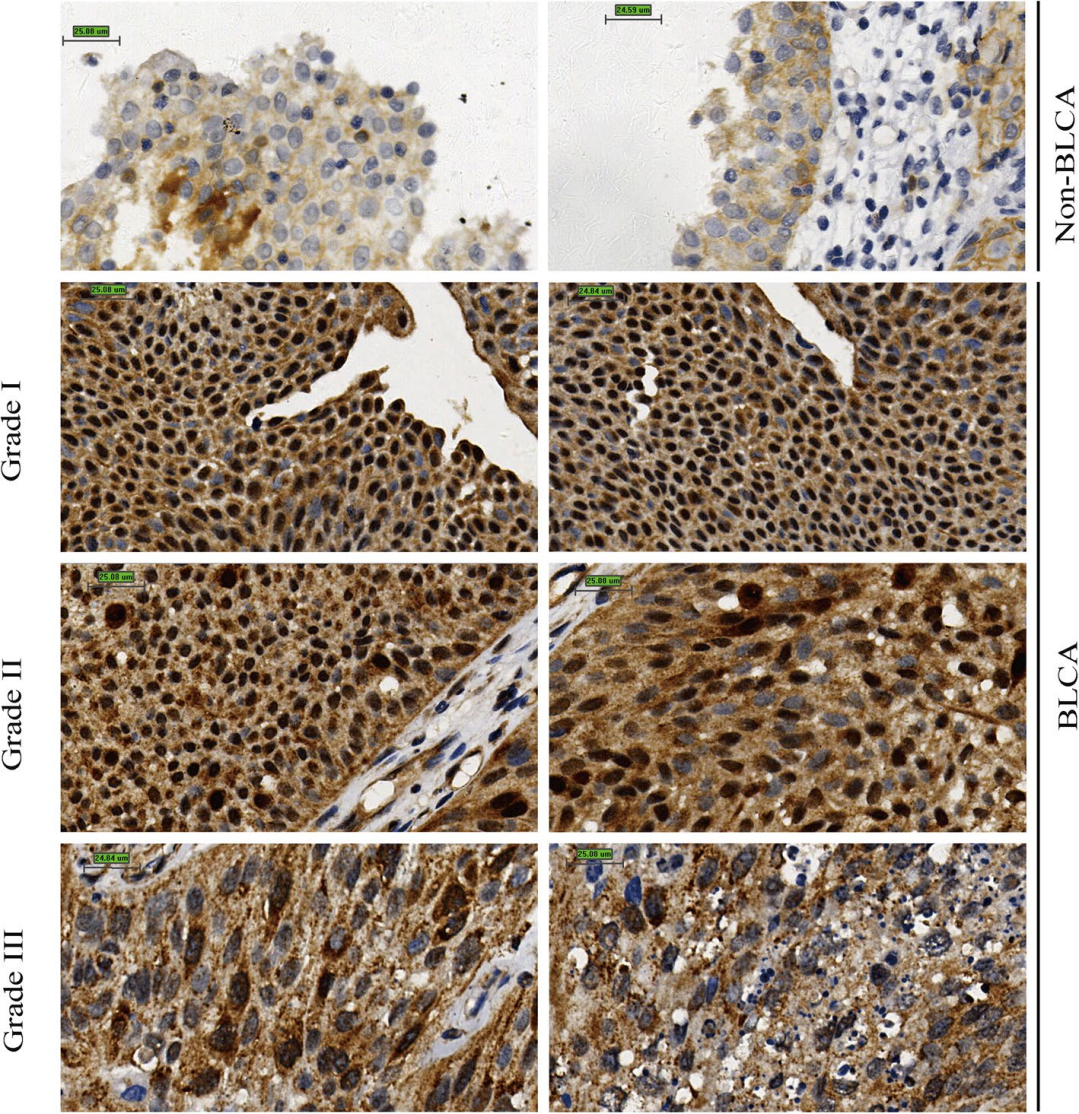
Over-expression of PTTG1 protein in bladder carcinoma tissue. In-house immunohistochemistry was conducted to explore the protein expression level of PTTG1, and PTTG1 protein was overexpressed in bladder carcinoma (BLCA) tissue samples when compared with non-BLCA tissue samples

**Fig. 2:**
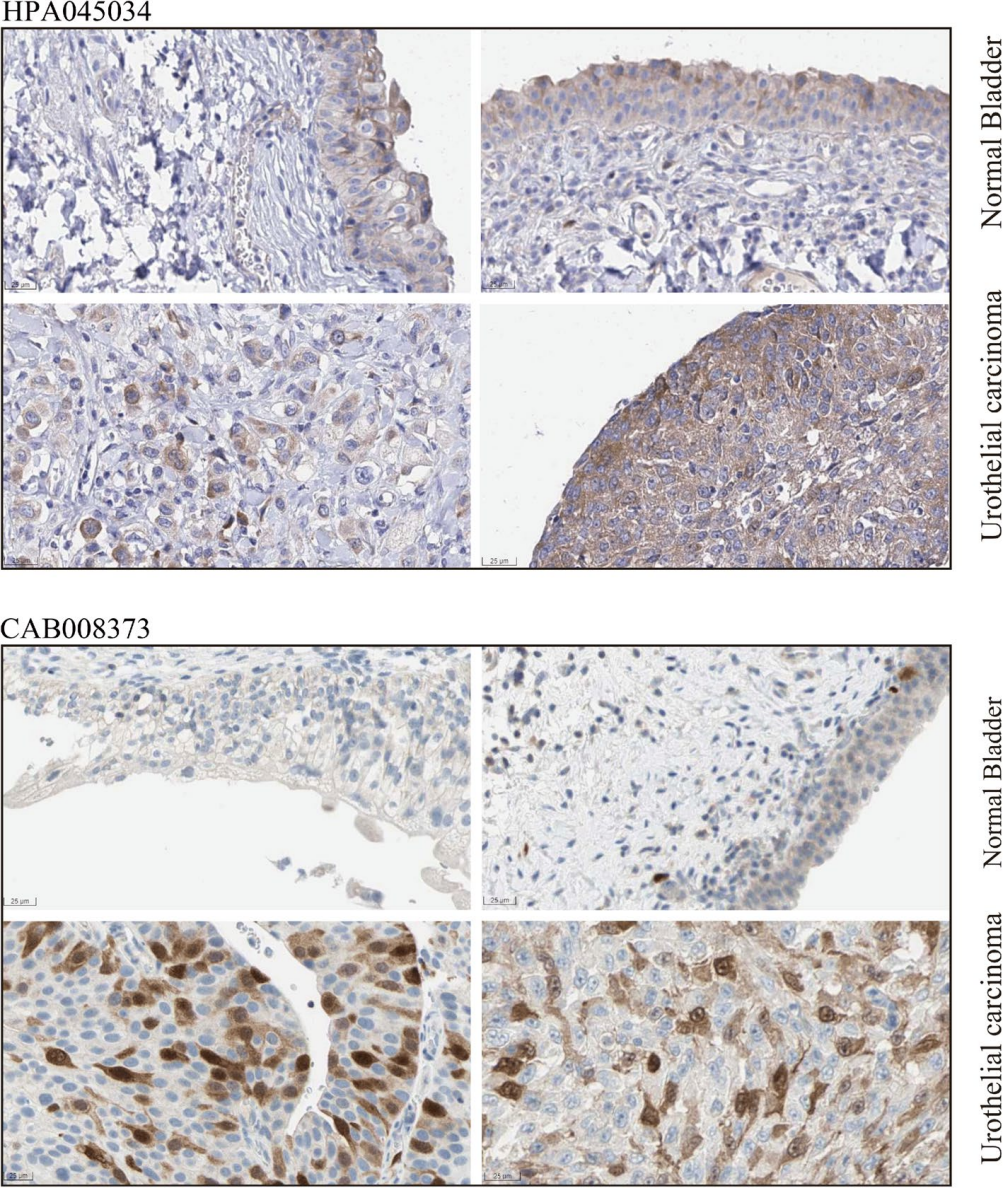
Over-expression of PTTG1 protein in the urothelial carcinoma of the bladder. The protein expression level of PTTG1 was confirmed by the immunohistochemistry result from the human protein atlas. PTTG1 protein was moderately stained in the urothelial carcinoma of the bladder and was lowly stained or not detected in normal or inflammatory bladder tissue (Antibody: HPA045034 and CAB008373)

A total of 47 BLCA and 10 non-BLCA tissue specimens were utilized to perform in-house immunohistochemical staining. PTTG1 antibody was strongly stained in BLCA tissue as opposed to non-BLCA control tissue (Fig. 1). Consistently, according to the immunohistochemical result from THPA, the PTTG1 antibody was moderately stained in the urothelial carcinoma of the bladder and was lowly stained or not detected in normal or inflammatory bladder tissue (Antibody: HPA045034 and CAB008373) (Fig. 2), which confirmed the elevated protein expression levels of PTTG1 in BLCA. Moreover, it was observed that PTTG1 localized predominantly in the cytoplasm and partially localized in the nucleus, which could be inferred from in-house immunohistochemistry and THPA results (https://www.proteinatlas.org/ENSG00000164611-PTTG1/subcellular).

**Fig. 3:**
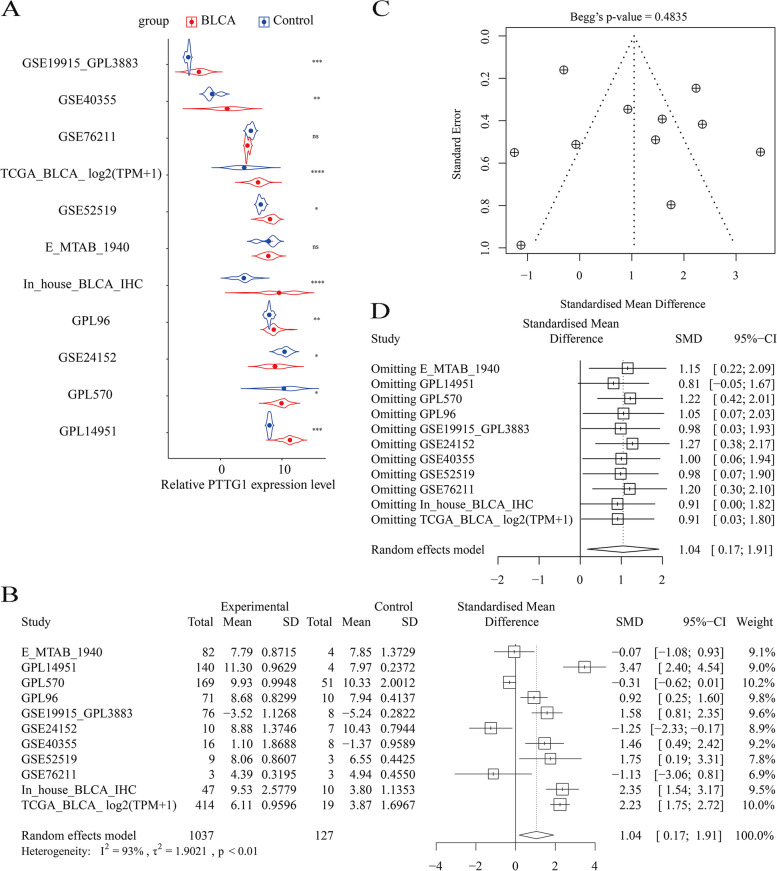
Significant over-expression of PTTG1 mRNA in bladder carcinoma tissue samples. **A** The transcriptome data from TCGA, GEO, and ArrayExpress implied an increased expression trend of PTTG1 mRNA in bladder carcinoma tissue samples. **B** PTTG1 was significantly up-regulated in global bladder carcinoma tissue specimens. **C** Begg’s funnel plot. Insignificant bias was detected (*P* > 0.05). **D** Forest plot of sensitive analysis. The standardized mean difference result may be unstable

Furthermore, the increased expression level of PTTG1 mRNA was supported by gene microarrays and mRNA sequencing data sets (Fig. 3A). The overall expression trend of PTTG1 in BLCA was assessed in global BLCA tissue samples. In general, the expression level of PTTG1 was significantly increased in 1037 BLCA tissue samples compared with 127 non-BLCA control tissue samples, with an SMD of 1.04 [0.17, 1.91] (Fig. 3B). No bias was observed (Fig. 3C). However, the forest plot result of sensitive analysis implied that the SMD result may be unstable (Fig. 3D). Therefore, more experimental verification is needed in the future.

**Fig. 4:**
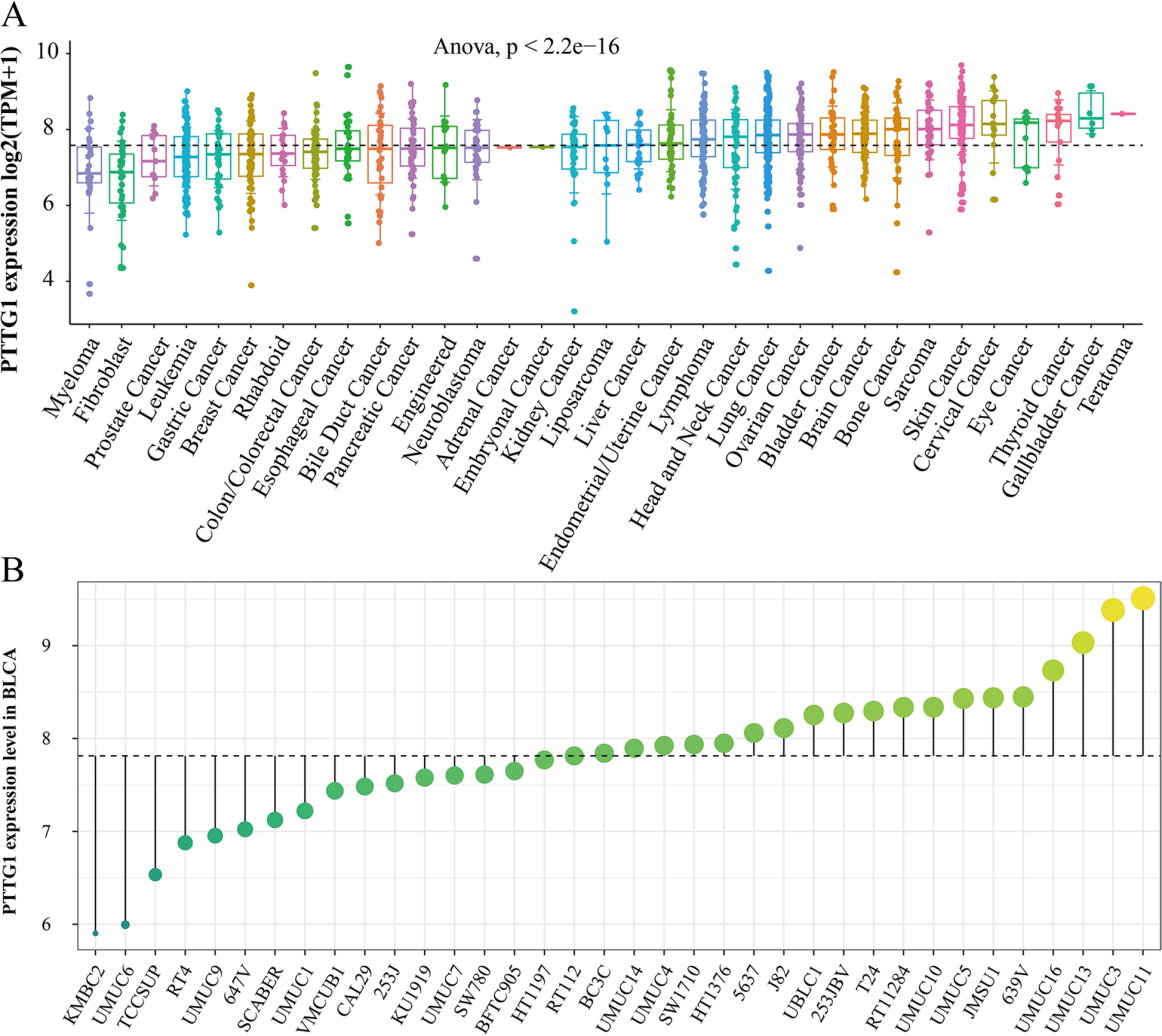
Over-expression verification of PTTG1 in bladder carcinoma cell lines. **A** The relative expression levels of PTTG1 mRNA were compared in pan-cancer cell lines. **B** PTTG1 mRNA was broadly expressed in a series of bladder carcinoma cell lines

As is shown in Fig. 4A, the relative expression levels of PTTG1 mRNA were also compared in pan-cancer cell lines. PTTG1 mRNA was broadly expressed in a series of BLCA cell lines (Fig. 4B). More importantly, the pro-tumor role of PTTG1 was preliminarily investigated in BLCA cell lines. As is shown in Fig. S1A, the CRISPR knockout Chronos gene effect scores of PTTG1 were all less than zero in BLCA cell lines, indicating that PTTG1 deletion may suppress the proliferation and survival of BLCA cells. Additionally, the RNAi knockout result also showed that PTTG1 was essential for the survival of BLCA cell lines (RNAi DEMETER2 score < 0, Fig. S1B).

### Potential clinical value of PTTG1 in BLCA patients

**Fig. 5:**
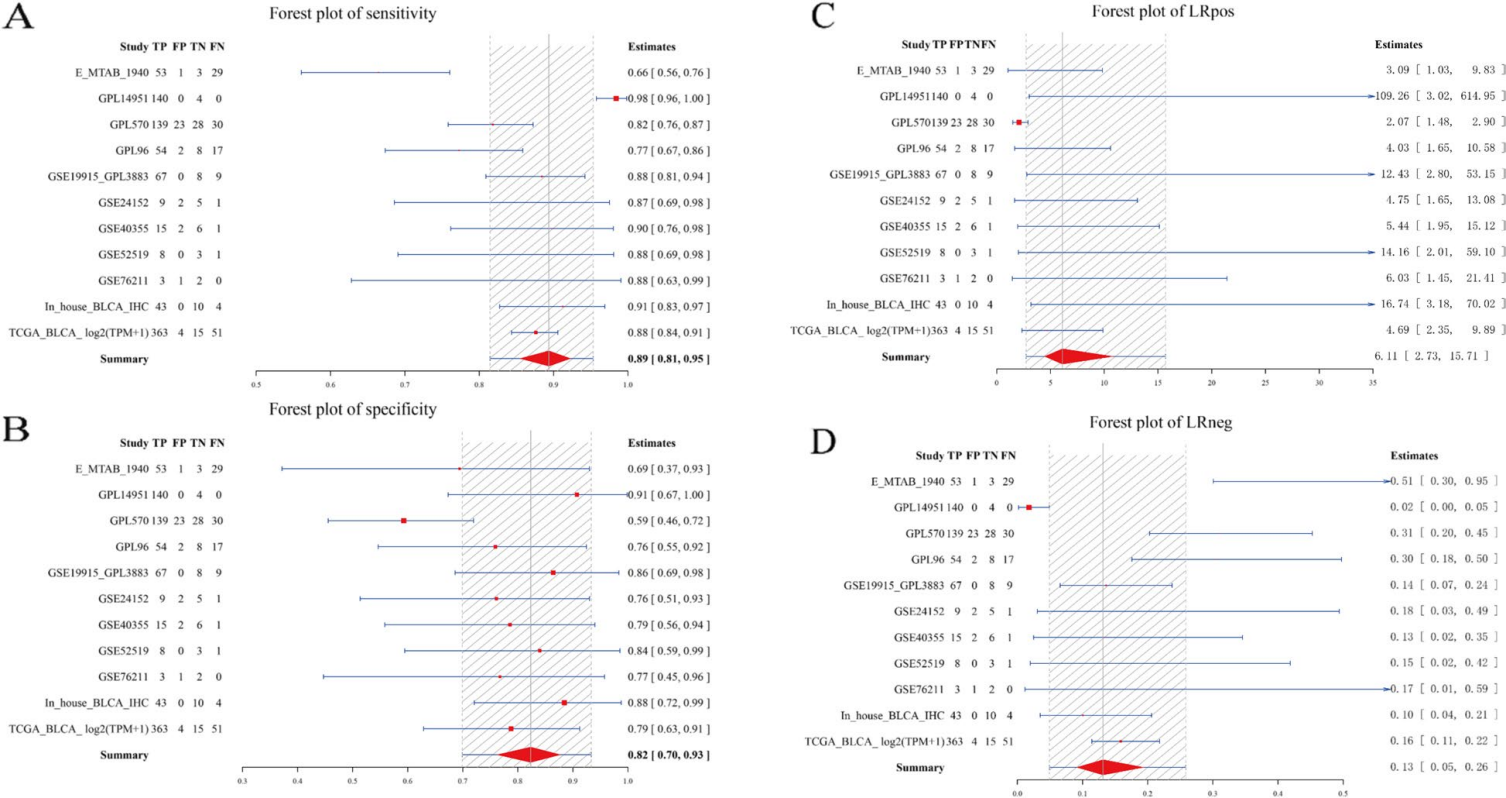
A strong discriminatory ability of PTTG1 in bladder carcinoma tissue samples. PTTG1 showed a strong discriminatory ability between bladder carcinoma tissue samples and normal bladder tissue samples, with a high sensitivity (**A**) and a high specificity (**B**). The accuracy of PTTG1 in differentiating bladder carcinoma from normal tissue samples was confirmed by a high positive likelihood ratio (**C**) and a low negative likelihood ratio (**D**)

**Fig. 6:**
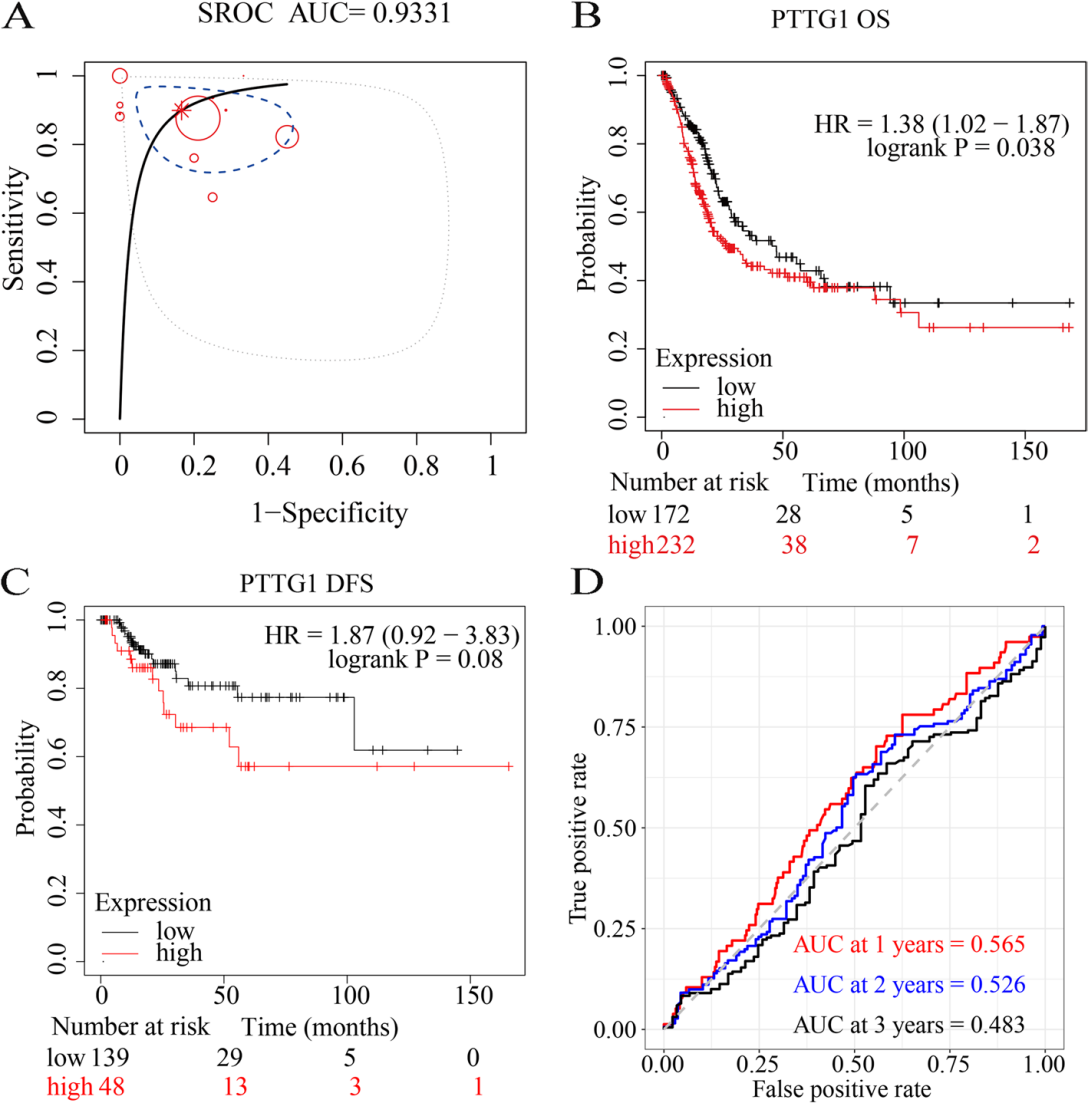
PTTG1 as an unfavorable prognostic biomarker in bladder carcinoma. A summary receiver operating characteristic curve was used to evaluate the comprehensive discriminatory ability of PTTG1 in bladder carcinoma. Intriguingly, PTTG1 displayed a strong ability in differentiating bladder carcinoma tissue samples from normal bladder tissue samples (AUC > 0.9) (**A**). Kaplan-Meier survival analysis was performed to determine the prognostic value of PTTG1 in bladder carcinoma. Higher PTTG1 mRNA levels presaged worse overall survival probability (HR > 1, *P* < 0.05) (**B**) and poorer disease-free survival outcomes (HR > 1, *P* > 0.05) (**C**). A time-dependent receiver operating characteristic curve was utilized to evaluate the 1-year, 2-year, and 3-year survival rates of bladder carcinoma patients (**D**)

In subsequent analysis, the authors explored the potential clinical implication of PTTG1 in BLCA patients. PTTG1 showed a strong discriminatory ability between BLCA tissue samples and normal bladder tissue samples (Fig. 5A–D, Fig. 6A), with an AUC value of 0.93, a high sensitivity (0.89 [0.81, 0.95]), and a high specificity (0.82 [0.70, 0.93]). The accuracy of PTTG1 in differentiating BLCA from normal tissue samples was confirmed by a high positive likelihood ratio (6.11 [2.73, 15.71]) and a low negative likelihood ratio (0.13 [0.05, 0.26]). Additionally, higher PTTG1 mRNA levels presaged worse OS probability in the TCGA-BLCA cohort (sample size: 404) (Fig. 6B). Although insignificant, higher PTTG1 mRNA levels tended to show poorer DFS outcomes (sample size: 187) (Fig. 6C). The result of the time-dependent receiver operating characteristic curve showed the weak prognostic ability of PTTG1 in the TCGA-BLCA cohort (Fig. 6D).

### Association between PTTG1 expression and BLCA TME

**Fig. 7:**
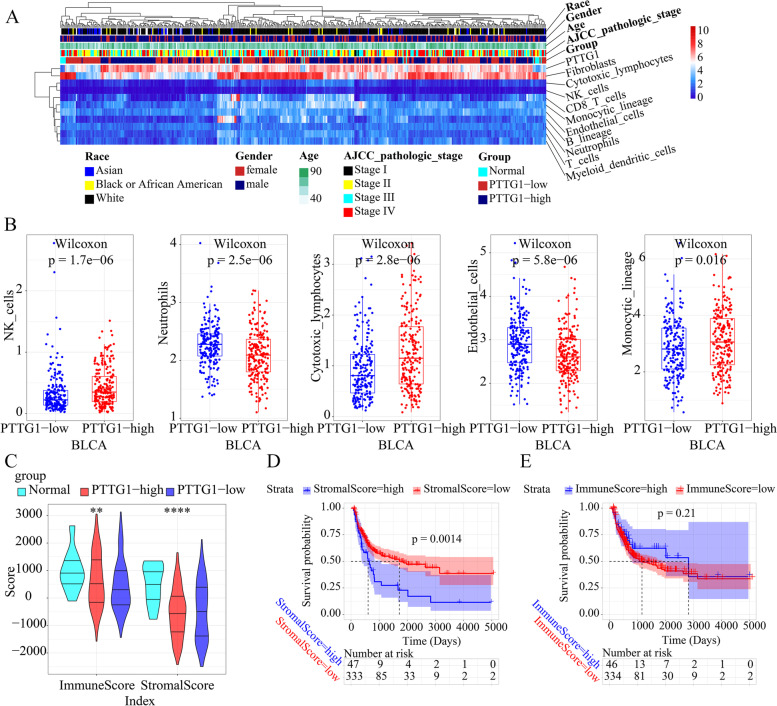
Potential association between PTTG1 and tumor microenvironment in bladder cancer tissue samples. The immune infiltration levels of immune and stromal cells of TCGA-BLCA patients were quantified by using the Microenvironment cell populations-counter algorithm. **A** The immune microenvironment and clinical phenotypes were compared in normal bladder tissues, PTTG1 low expression bladder cancer tissue samples, and PTTG1 high expression bladder cancer tissue samples. **B** Higher PTTG1 mRNA expression levels indicated higher natural killer, cytotoxic lymphocyte, and monocyte lineage cell infiltration levels, and indicated lower neutrophils and endothelial cell infiltration levels. **C** The immune score was significantly lower in low PTTG1 mRNA expression bladder cancer tissue samples than that in high PTTG1 mRNA expression bladder cancer tissue samples or normal bladder tissue samples. However, no difference was detected in the stromal score of low PTTG1 mRNA expression bladder cancer tissue samples than that in high PTTG1 mRNA expression bladder cancer tissue samples (*P* < 0.05). **D** A high stromal score predicted poor prognosis in bladder carcinoma patients. **E** The prognostic value of the immune score in bladder carcinoma was insignificant (P < 0.05). **, *P* < 0.01; ****, *P* < 0.0001

As is shown in Fig. 7A, the author first compared the immune microenvironment and clinical phenotypes among normal bladder tissue, PTTG1 low expression BLCA tissue, and PTTG1 high expression BLCA tissue samples. It was observed that higher PTTG1 mRNA expression levels indicated higher natural killer (NK), cytotoxic lymphocyte, and monocyte lineage cell infiltration levels, and it indicated lower neutrophils and endothelial cell infiltration levels (Fig. 7B). Additionally, according to the result of TIMER, the PTTG1 expression level positively correlated to the infiltration degrees of CD8^+^ T cells (R = 0.302, *P* = 3.64e− 9) and dendritic cells (R = 0.416, *P* = 1.12e− 16) (Fig. S2). The immune score was significantly lower in low PTTG1 mRNA expression BLCA tissue samples than that in high PTTG1 mRNA expression BLCA tissue samples or normal bladder tissue samples (Fig. 7C). However, no difference was detected in the stromal score of low PTTG1 mRNA expression BLCA tissue samples than that in high PTTG1 mRNA expression BLCA tissue samples (Fig. 7C). Moreover, a high stromal score predicted a poor prognosis in BLCA patients (Fig. 7D). The prognostic value of the immune score in BLCA patients was insignificant (Fig. 7E).

### Prospective transcriptional mechanisms of PTTG1 in BLCA at the single-cell resolution

**Fig. 8:**
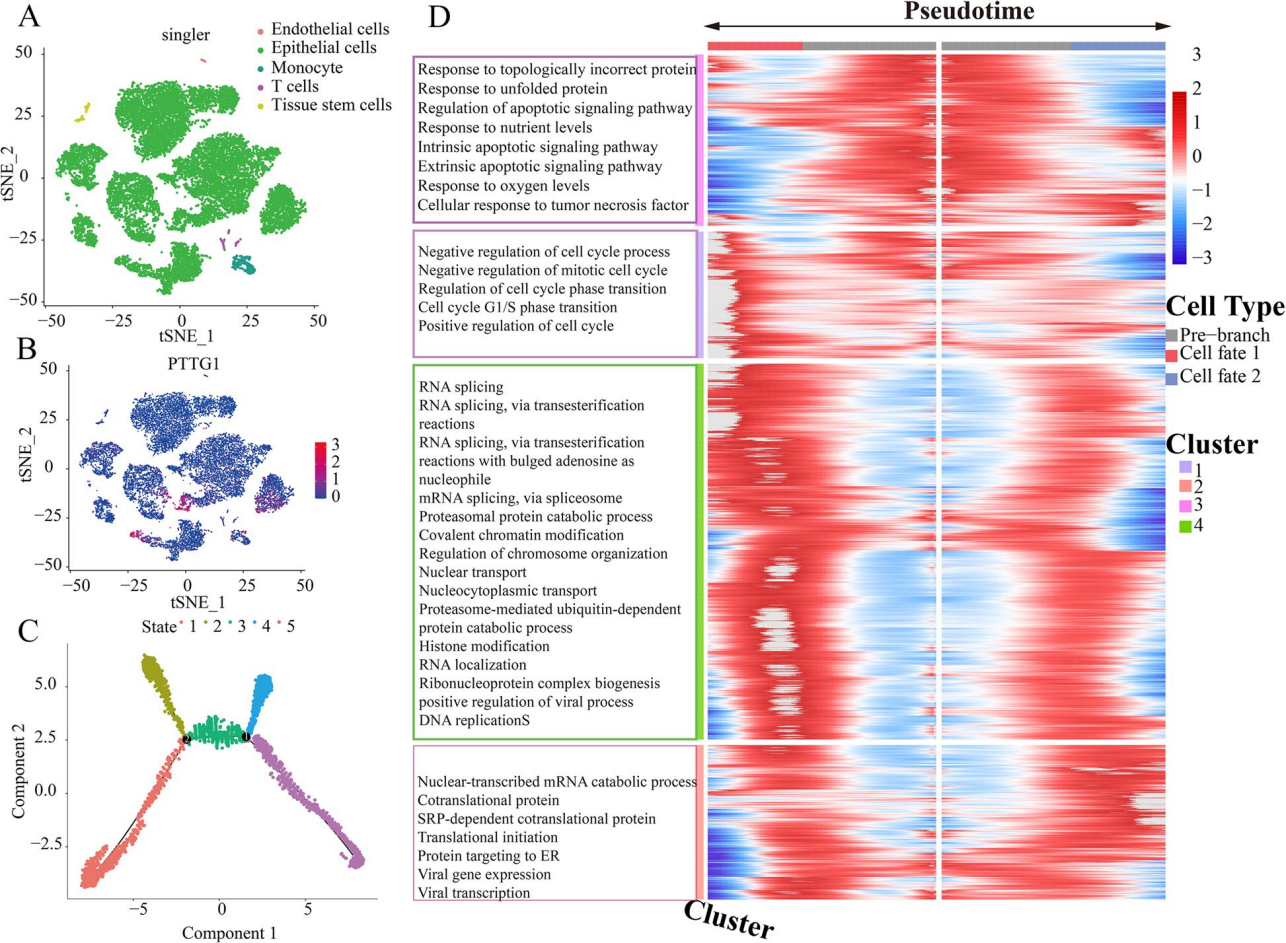
Potential transcriptional regulatory mechanisms of PTTG1 in bladder cancer at single-cell resolution. GSE135337 dataset was utilized for analyzing the transcriptional regulatory mechanisms of PTTG1 in seven bladder cancer patients. Single cells from bladder cancer patients were clustered and annotated. Epithelial cells were the predominant cell type (**A**). The expression distributions of PTTG1 were shown in single cells. PTTG1 was predominantly expressed in cancerous epithelial cells (**B**). **C** Epithelial cells from bladder cancer tissues were filtered for pseudotime analysis. **D** Gene expression heatmap along the pseudotime direction. The gene expression alterations at the first branch were analyzed and the differentially expressed genes were functionally annotated. Cell fate 1 and cell fate 2 in panel D refer to state 4 and state 5 in panel C, respectively

Given the intimate association between PTTG1 and BLCA, the author subsequently explored its potential transcriptional mechanisms underlying BLCA. Single cells from seven BLCA patients were clustered and annotated by using GSE135337 scRNA-seq profile, where cancerous epithelial cells were the predominant cell types (Fig. 8A). We tried to identify the cell distribution of PTTG1 expression, and it was found that PTTG1 was mainly expressed in the epithelial cells (Fig. 8B). Next, epithelial cells from BLCA tissues were subsetted for performing pseudotime analysis, through which we could infer the cell fate of cancerous cells and remodel the process of cell changes over time (Fig. 8C). Figure 8D showed the gene expression heatmap in the epithelial cells along the pseudotime direction. The cell fate-related DEGs were functionally clustered in the negative regulation of cell cycle process, nuclear-transcribed mRNA catabolic process, nonsense-mediated decay, response to topologically incorrect protein, and RNA splicing.

**Fig. 9:**
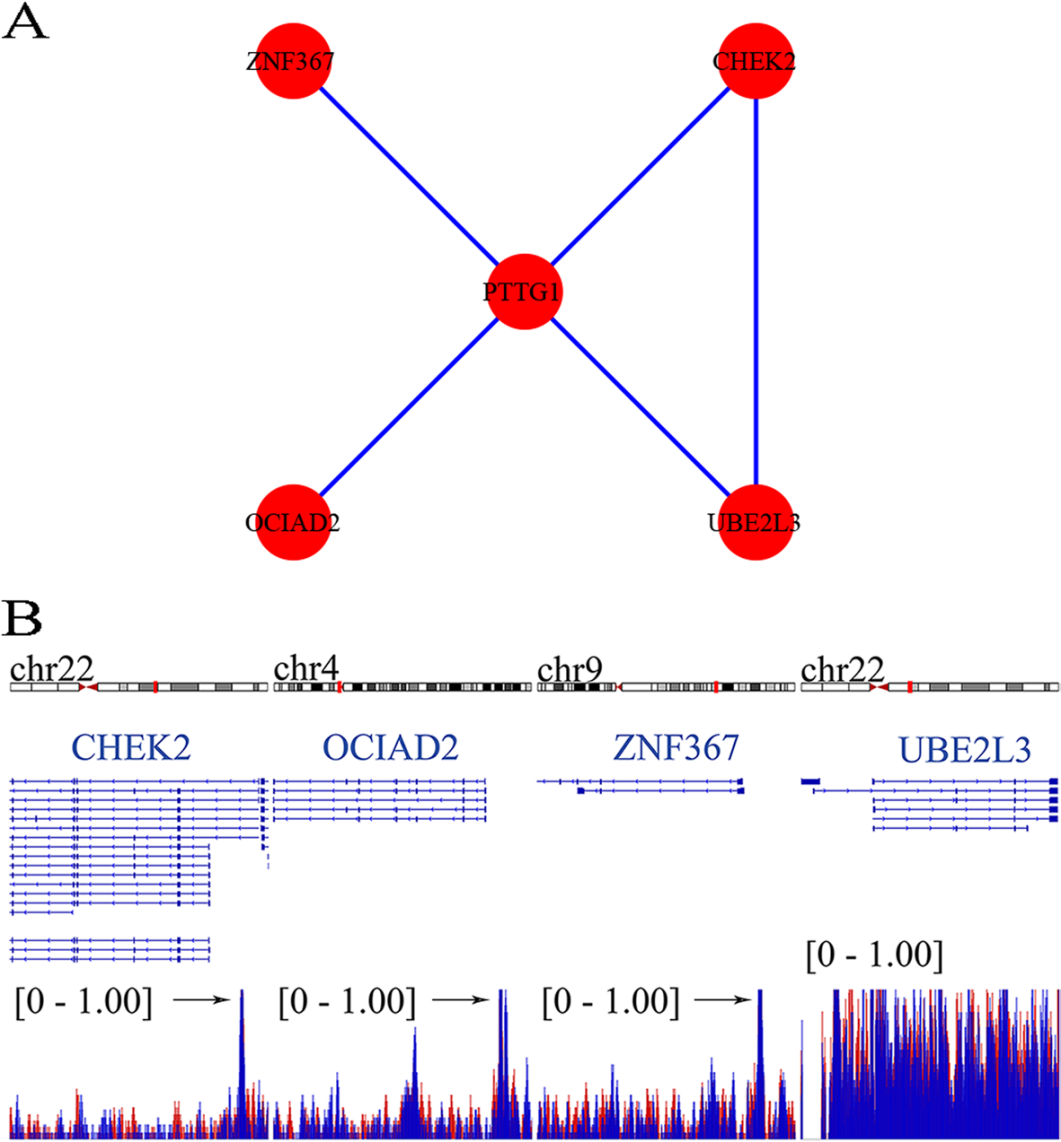
Transcriptional targets of PTTG1 were identified by using chromatin immunoprecipitation followed by sequencing data from CistromeDB. **A** The interplay between the PTTG1 transcriptional factor and four transcriptional targets was analyzed in a protein-protein interaction network. **B** Chromatin immunoprecipitation followed by sequencing data was used to explore the binding peak of PTTG1 in the promoter regions of transcriptional targets. *CHEK2*, *OCIAD2*, *UBE2L3*, and *ZNF367* were predicted as transcriptional targets of PTTG1 in bladder carcinoma. The data were downloaded from CistromeDB (ID: 63264 and 63265)

**Fig. 10:**
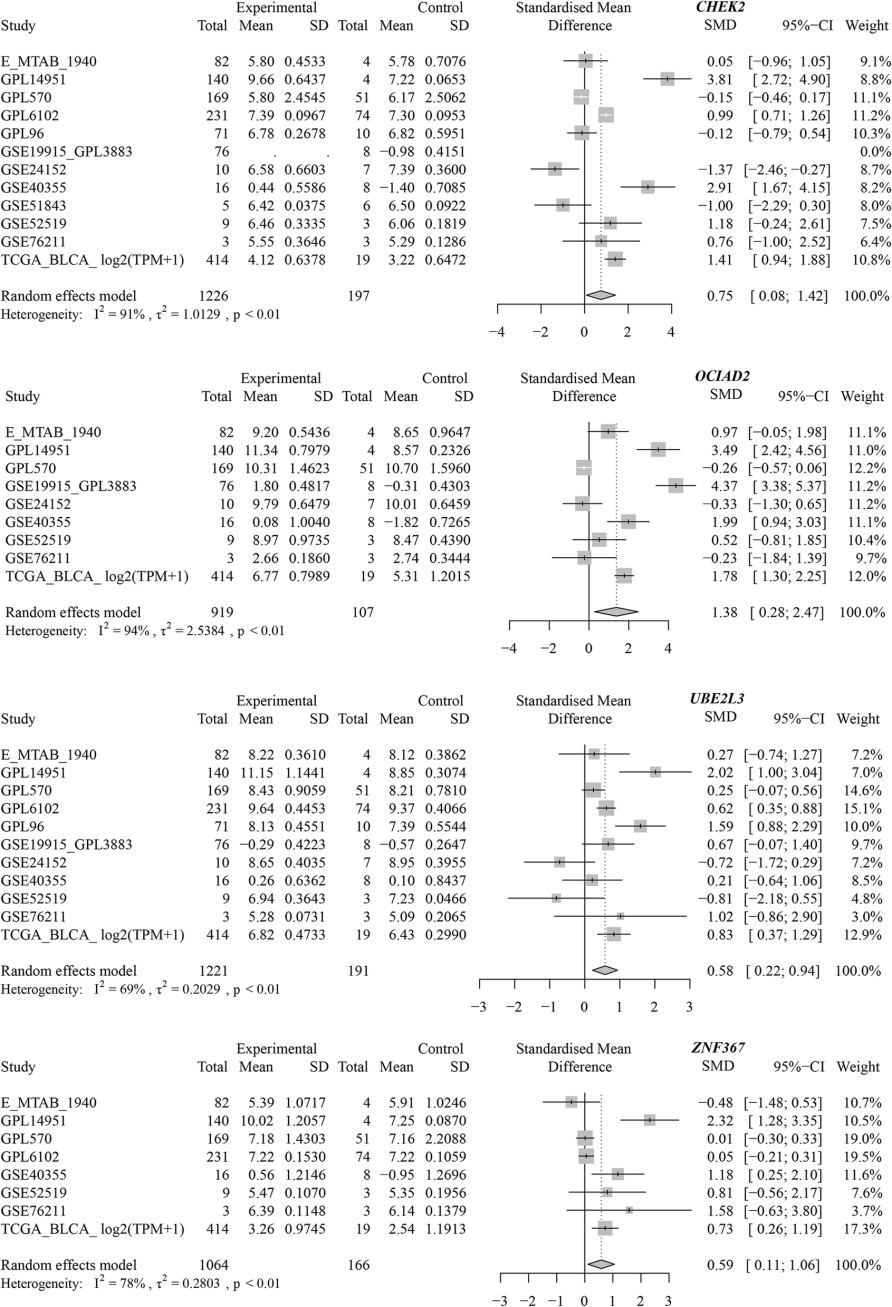
Global expression trends of PTTG1 transcriptional targets in BLCA tissue. *CHEK2*, *OCIAD2*, *UBE2L3*, and *ZNF367* were significantly up-regulated in BLCA tissue samples. BLCA, bladder carcinoma

Finally, a total of 73 genes were preserved by intersecting cell fate-related DEGs, BLCA DEGs, and putative TF targets. By studying the protein-protein interaction network, *CHEK2* (checkpoint kinase 2), *OCIAD2* (OCIA domain containing 2), *UBE2L3* (ubiquitin-conjugating enzyme E2 L3), and *ZNF367* (zinc finger protein 367) were predicted to be the transcriptional targets of PTTG1 in BLCA (Fig. 9A). More importantly, ChIP-seq data were used to explore the binding peak of PTTG1 in the promoter regions of such four targets (Fig. 9B), indicating the transcriptional regulatory relationship between them. We also preliminarily validated the transcriptional targets of PTTG1 in BLCA by differential and co-expression analysis. As is shown in Fig. S3, *CHEK2*, *OCIAD2*, *UBE2L3*, and *ZNF367* all displayed increased expression trends in the TCGA-BLCA cohort. Moreover, the over-expression of such targets was certified by the global BLCA mRNA datasets (*CHEK2*: SMD =0.75; *OCIAD2*: SMD =1.38; *UBE2L3*: SMD =0.58; *ZNF367*: SMD =0.59) (Fig. 10). Furthermore, there was a significant positive correlation between PTTG1 and such four targets (*CHEK2*: Spearman R = 0.44; *OCIAD2*: Spearman R = 0.32; *UBE2L3*: Spearman R = 0.37; and *ZNF367*: Spearman R = 0.61), thus suggesting that PTTG1 may positively regulate the transcriptional activity of *CHEK2*, *OCIAD2*, *UBE2L3*, and *ZNF367* targets.

**Fig. 11:**
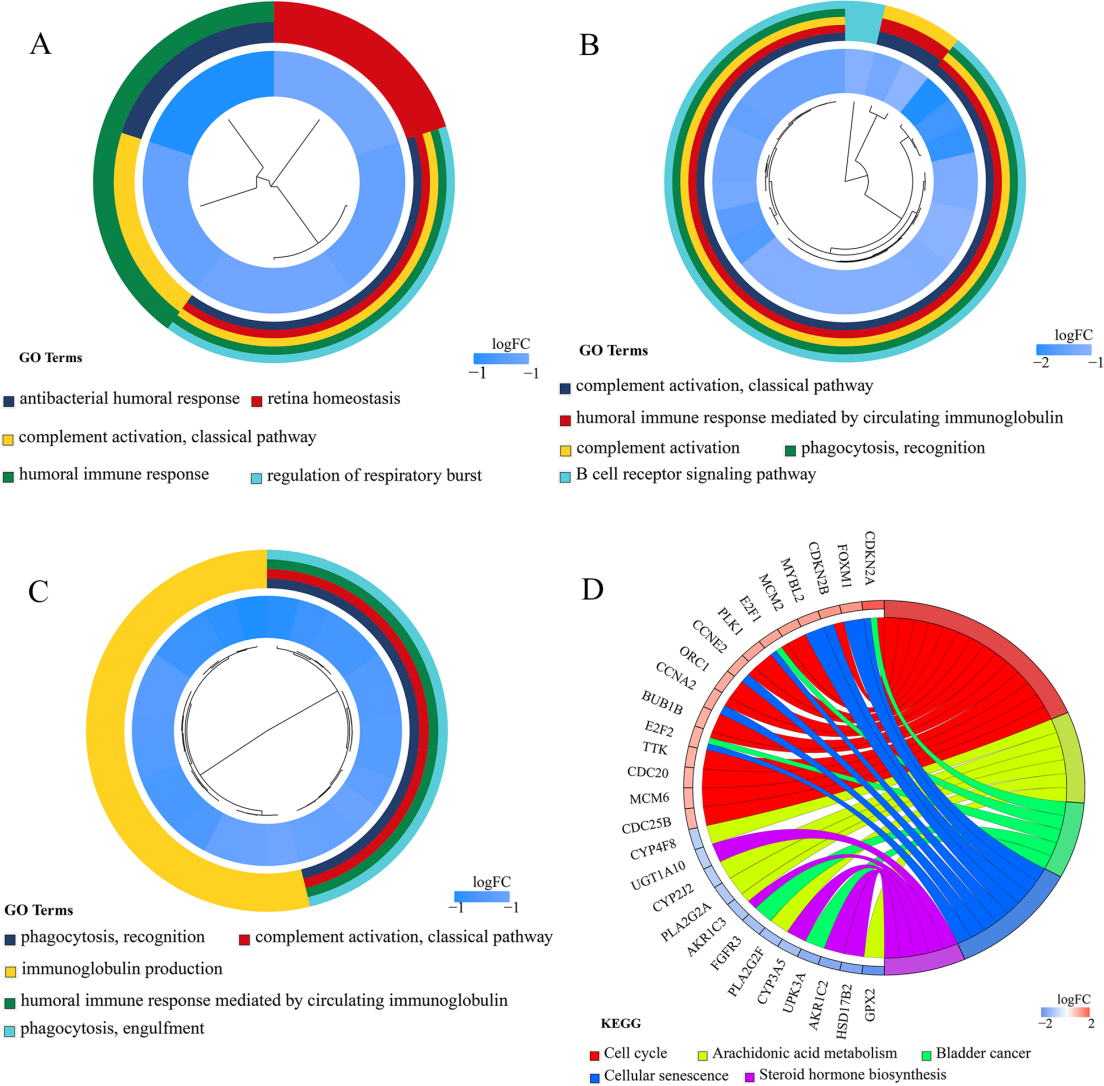
Functional enrichment of PTTG1 transcriptional targets in BLCA tissue. Differentially expressed genes (DEGs) associated with *CHEK2*, *OCIAD2*, *UBE2L3*, and *ZNF367* were identified from the TCGA-BLCA cohort study. The prospective biological functions of these PTTG1 targets were annotated. A. *CHEK2*-related DEGs were significantly enriched in the antibacterial humoral response. B. *OCIAD2*-related DEGs were significantly enriched in the complement activation (classical pathway). C. *UBE2L3*-related DEGs were significantly enriched in the phagocytosis and recognition. D. *ZNF367*-related DEGs were significantly enriched in the cell cycle pathway. BLCA, bladder carcinoma

Intriguingly, in-depth functional enrichment result indicated that PTTG1 played different roles by targeting *CHEK2*, *OCIAD2*, *UBE2L3*, and *ZNF367* genes. It was observed that *CHEK2*, *OCIAD2*, and *UBE2L3*-related genes were significantly accumulated in the humoral immune response (Fig. 11A–C). Regarding *ZNF367*-related genes, cell cycle, arachidonic acid metabolism, and bladder cancer were the predominantly enriched pathways (Fig. 11D). Therefore, PTTG1 targeting *CHEK2*, *OCIAD2*, *UBE2L3*, and *ZNF367* genes may participate in the development of BLCA.

The author also evaluated the BLCA prognosis prediction implication of PTTG1 methylation level subsequently. Intriguingly, high methylation levels of PTTG1, as well as its potential transcriptional targets (i.e., *CHEK2*, *OCIAD2*, *UBE2L3*, and *ZNF367*), could predict the poor prognosis of BLCA patients preferably (*P* < 0.05) (Figs. S4–S7). Among them, the methylation levels of *OCIAD2*, *UBE2L3*, and *ZNF367* were negatively correlated to their mRNA expression levels, accordingly (*OCIAD2*: Spearman R = − 0.33; *UBE2L3*: Spearman R = − 0.47; and *ZNF367*: Spearman R = − 0.23). Taken together, the methylation levels of PTTG1, as well as its targets, may be useful in the prognosis stratification of BLCA patients.

### Promising small molecules for BLCA treatment

As is shown in Table 1, several perturbations were identified by targeting the transcriptional regulatory network of PTTG1. Among them, formestane has been put into use in the clinical treatment of breast cancer patients [31, 32]. Additionally, reparixin could disrupt the cancer stem cell properties in oral squamous cell carcinoma [33]. In this setting, our study may provide a novel direction for treating BLCA cells by drug repurposing analysis. However, more experimental verification is needed.

**Table 1:** Potential small molecules for treating bladder cancer cells by targeting the PTTG1 transcriptional regulatory network

Perturbagen	Cell line	Dose	Time	Sample	Description	Target	Raw CS	Normalized CS	−log_**10**_q
MAP3K9	AALE	/	96 h	3	/	/	−0.59	−2.14	15.65
TGFA	HA1E	10 ng/ml	4 h	3	/	/	−0.58	−2.12	15.65
MAX	SALE	/	96 h	3	/	/	−0.59	−2.12	15.65
Reparixin	HT29	0.03 μM	24 h	2	CC chemokine receptor antagonist	CXCR1|CXCR2	−0.58	−2.1	15.65
RELB	HT29	/	96 h	3	/	/	−0.58	−2.09	15.65
KIAA0494	HT29	1 μL	96 h	3	/	/	−0.58	−2.09	15.65
OTUD7A	HT29	1 μL	96 h	3	/	/	−0.58	−2.09	15.65
XPO7	HT29	/	96 h	2	/	/	−0.57	−2.08	15.65
IL1RAP	A375	/	96 h	2	/	/	−0.57	−2.07	15.65
BRD-A47816767	HT29	10 μM	24 h	3	/	/	−0.57	−2.07	15.65
BRD-K51126483	VCAP	10 μM	6 h	4	/	/	−0.57	−2.06	15.65
BRD-K36772364	HCC515	10 μM	6 h	3	/	/	−0.57	−2.06	15.65
Formestane	A375	10 μM	6 h	3	Selectively steroidal aromatase inhibitor (type I)	/	−0.57	−2.06	15.65
BRD-K68657207	VCAP	4 μM	24 h	3	/	/	−0.57	−2.05	15.65
TCEAL4	MCF7	/	96 h	2	/	/	−0.56	−2.04	15.65

*CS* connectivity score

## Discussion

Herein, the authors investigated the global expression status and prospective transcriptional regulation mechanisms of PTTG1 in BLCA. PTTG1 was significantly over-expressed in 1037 BLCA tissue samples and showed a strong ability in distinguishing BLCA tissue from normal bladder tissue. High PTTG1 expression showed increased antitumor activity in BLCA tissues, with elevated infiltration levels of NK, cytotoxic lymphocytes, and monocytic lineage cells. More importantly, the authors preliminarily identified the positive transcriptional activity between PTTG1 and *CHEK2*, *OCIAD2*, *UBE2L3*, and *ZNF367*.

The over-expression trend of PTTG1 in BLCA was certified using multi-faceted data sets. To probe the comprehensive expression status of PTTG1 in BLCA tissue samples, we made full use of the in-house immunohistochemistry data and external expression matrices, and a total of 1037 BLCA tissue specimens, as well as 127 normal bladder tissue specimens, were integrated. Consistently, we observed the increased expression trends of PTTG1 at both tissue and cell levels. Additionally, PTTG1 mRNA may be a strong distinguishing biomarker for BLCA and its over-expression could presage poor OS conditions in BLCA patients. Moreover, the methylation level of PTTG1 could be used to forecast the poor prognosis of BLCA patients. In summary, it is suggested that PTTG1 mRNA over-expression may operate as a cancer-promoting factor and could have the potential to be a predictor of poor prognosis in BLCA.

Furthermore, the potential PTTG1 activity in BLCA TME was investigated. As is well known, the abnormal TME is a hotbed for cancer initiation and progression [34], where immune and stromal cells take an important part [35]. Among them, the mononuclear phagocyte system constitutes an essential part of human tumor immunity; and cytotoxic lymphocytes, together with NK cells, constitute an important defense line in anti-tumor immunity [36]. A previous study reported that higher PTTG1 expression could be found in the CD_4_^+^ and CD_8_^+^ T lymphocytes of adult T-cell leukemia patients than that of healthy subjects [37], which implied an intimate association between PTTG1 and lymphocytes. According to *Rostyslav Stoika* et al., the mRNA abundance of PTTG1 corresponded to the increase in S-phase cells during the activation of T lymphocytes [38], which suggested that PTTG1 expression may follow the cell cycling patterns in T cells. In the present study, a high PTTG1 mRNA expression group was shown to be enriched with NK, cytotoxic lymphocyte, and monocyte lineage cells in BLCA patients. Furthermore, the immune score was significantly higher in the high PTTG1 mRNA expression group than that in the low PTTG1 mRNA expression group in BLCA tissue samples. Moreover, there was a positive association between the mRNA expression levels of PTTG1 and dendritic cell infiltration levels, which reflexed the antigen-presenting activity in BLCA tissue. In this setting, we inferred that PTTG1 may serve as an oncogene and possess immunogenicity in BLCA, which has the potential to be designed as an mRNA vaccine in the future. Intriguingly, it has been shown that SP17/AKAP4/PTTG1 could induce an immunogenic response in non-small cell lung cancer patients [39]. Moreover, a high expression level of PTTG1 was correlated to the immune checkpoint response in the papillary renal cell carcinoma cohort [40]. Such evidence pointed out that PTTG1 may be a promising immunotherapeutic target for BLCA patients. More experiments are required to be performed to promote the research of novel cancer vaccines for BLCA in future studies.

The prospective transcriptional mechanisms of PTTG1 were preliminarily portrayed in BLCA. *CHEK2*, *OCIAD2*, *UBE2L3*, and *ZNF367* were predicted to be four positive transcriptional targets of PTTG1 in BLCA. Multiple studies have reported these four transcriptional targets of PTTG1 in tumor tissue. For instance, researchers performed immunohistochemical staining in BLCA samples and showed that CHEK2 protein expression surpassed 11% in 115 out of 126 BLCA tissue specimens [41]. Additionally, *CHEK2* mutation was reported to be a risk factor for the recurrence of BLCA [42]. Similarly, *OCIAD2*, *UBE2L3*, and *ZNF367* also displayed intimate association with cancer development. In previous function assays, *OCIAD2* was shown to be essential for the activation of signal transducer and activator of transcription 3 and cell migration, which suggested that *OCIAD2* may contribute to the metastasis of cancer cells [43]. In liver cancer and oral squamous cell carcinoma, *UBE2L3* was reported to be an important pro-tumorigenic factor in carcinogenesis [44, 45] and may be a potential treatment target for hepatocellular carcinoma [46]. Moreover, *ZNF367* could induce the transcriptional activation of kinesin family member 15, leading to elevated cell viability and invasion ability in breast cancer cells [47]. Nonetheless, the biological functions of *OCIAD2*, *UBE2L3*, and *ZNF367* in BLCA remained obscure, and more experimental exploration is required to understand their roles in BLCA development. Generally, the predicted transcriptional mechanism results supported the previous speculation that over-expression of PTTG1 seemed to be associated with the initiation and development of BLCA.

However, as is implicated by the in-house immunohistochemistry and THPA results, the gene product of PTTG1 belongs to a cytosolic protein, although it is partially localized in the nucleus; this raised an interesting question that how PTTG1 could regulate the transcription of its targeted genes. Surprisingly, as a global transcriptional factor, PTTG1 could, directly and indirectly, induce the expression of genes and promote tumor development [48]. For instance, PTTG1 could bind to the c-Myc promoter region and activate c-Myc oncogene transcription, which resulted in cellular transformation and tumorigenesis [49, 50]. Additionally, PTTG1 also interacted with the other transcription factors. It was confirmed that PTTG1 interacted with the p53 transcription factor and inhibited its specific binding to DNA, thus blocking the transcriptional activity of the p53 tumor suppresser gene [51]. Moreover, PTTG1 functioned coordinately with Sp1 and up-regulated cyclin D3, promoting G1/S phase transition [52]. In this context, PTTG1 may regulate the transcription of targeted genes by direct and indirect interaction. In the future, it is important to investigate whether there is any activator/ligand or signaling mechanism translocating the cytoplasm PTTG1 to the nucleus.

The present study has numerous highlights. Using integrated in-house immunohistochemical data from our institution, TCGA, ArrayExpress, and GEO datasets, we fully discovered PTTG1 over-expression in BLCA and its potential as a biomarker and prognostic value. Our study may reveal a novel direction for the transcriptional mechanism investigation of PTTG1 in BLCA. The limitations of this study could not be overlooked. First, even though we combined datasets from multiple sources, the high-degree heterogeneity could not be eliminated by the randomized effect model. Second, the total sample size was limited, and more experimental studies must be carried out in the future to functionally validate the oncogene role of PTTG1 in BLCA. More evidence should be supplemented in future research to certify the transcriptional roles of PTTG1 in BLCA.

## Conclusions

PTTG1 mRNA over-expression may become a potential biomarker for BLCA prognosis. Additionally, PTTG1 may correlate with the BLCA tumor microenvironment and exert transcriptional activity by targeting *CHEK2*, *OCIAD2*, *UBE2L3*, and *ZNF367* in BLCA tissue.

## Supplementary Information

**Additional file 1.**

## Data Availability

The datasets generated and/or analyzed during the current study are available in Gene Expression Omnibus [https://www.ncbi.nlm.nih.gov/geo/], ArrayExpress [https://www.ebi.ac.uk/arrayexpress/], the Cancer Genome Atlas [https://portal.gdc.cancer.gov/], Cancer Dependency Map [https://depmap.org/portal/]. Kaplan-Meier Plotter is publicly available at http://kmplot.com/analysis/.

## Abbreviations

PTTG1: Pituitary tumor transforming gene-1
BLCA: Bladder cancer
GEO: Gene expression omnibus
TCGA: The cancer genome atlas
GTEx: Genotype-tissue expression
TF: Transcriptional factor
THPA: The human protein atlas
Depmap: Cancer dependency map
CRISPR: Clustered regularly interspaced short palindromic repeats
DEGs: Differently expressed genes
SMD: Standardized mean difference
OS: Overall survival
DFS: Disease-free survival
TME: Tumor microenvironment
FPKM: Fragments per kilobase of transcript per million fragments mapped
TPM: Transcript per million
scRNA-seq: Single-cell RNA sequencing
PC: Principal component
tSNE: t-distributed stochastic neighbor embedding
ChIP-seq: Chromatin immunoprecipitation followed by sequencing
AUC: Area under the curve
NK: Natural killer
CHEK2: Checkpoint kinase 2
OCLAD2: OCTA domain containing 2
UBE2L3: Ubiquitin-conjugating enzyme E2 L3
ZNF367: Zinc finger protein 367

## References

[1] Sung H, Ferlay J, Siegel RL, Laversanne M, Soerjomataram I, Jemal A (2021). Global Cancer Statistics 2020: GLOBOCAN Estimates of Incidence and Mortality Worldwide for 36 Cancers in 185 Countries. CA Cancer J Clin.

[2] Xia C, Dong X, Li H, Cao M, Sun D, He S (2022). Cancer statistics in China and United States, 2022: profiles, trends, and determinants. Chin Med J.

[3] Kamat AM, Hahn NM, Efstathiou JA, Lerner SP, Malmström PU, Choi W (2016). Bladder cancer. Lancet (London, England).

[4] Berdik C (2017). Unlocking bladder cancer. Nature..

[5] Ploussard G, Shariat SF, Dragomir A, Kluth LA, Xylinas E, Masson-Lecomte A (2014). Conditional survival after radical cystectomy for bladder cancer: evidence for a patient changing risk profile over time. Eur Urol.

[6] Pei L, Melmed S (1997). Isolation and characterization of a pituitary tumor-transforming gene (PTTG). Mol Endocrinol (Baltimore, Md).

[7] Cho-Rok J, Yoo J, Jang YJ, Kim S, Chu IS, Yeom YI (2006). Adenovirus-mediated transfer of siRNA against PTTG1 inhibits liver cancer cell growth in vitro and in vivo. Hepatology (Baltimore, Md).

[8] Fu D, Zhang Y, Cui H (2018). Long noncoding RNA CCAT2 is activated by E2F1 and exerts oncogenic properties by interacting with PTTG1 in pituitary adenomas. Am J Cancer Res.

[9] Tsai SJ, Lin SJ, Cheng YM, Chen HM, Wing LY (2005). Expression and functional analysis of pituitary tumor transforming gene-1 [corrected] in uterine leiomyomas. J Clin Endocrinol Metab.

[10] Solbach C, Roller M, Fellbaum C, Nicoletti M, Kaufmann M (2004). PTTG mRNA expression in primary breast cancer: a prognostic marker for lymph node invasion and tumor recurrence. Breast (Edinburgh, Scotland).

[11] Parte S, Virant-Klun I, Patankar M, Batra SK, Straughn A, Kakar SS (2019). PTTG1: a Unique Regulator of Stem/Cancer Stem Cells in the Ovary and Ovarian Cancer. Stem Cell Rev Rep.

[12] Repo H, Gurvits N, Löyttyniemi E, Nykänen M, Lintunen M, Karra H (2017). PTTG1-interacting protein (PTTG1IP/PBF) predicts breast cancer survival. BMC Cancer.

[13] Zou H, McGarry TJ, Bernal T, Kirschner MW (1999). Identification of a vertebrate sister-chromatid separation inhibitor involved in transformation and tumorigenesis. Science (New York, NY).

[14] Heaney AP, Nelson V, Fernando M, Horwitz G (2001). Transforming events in thyroid tumorigenesis and their association with follicular lesions. J Clin Endocrinol Metab.

[15] Ren Q, Jin B (2017). The clinical value and biological function of PTTG1 in colorectal cancer. Biomed Pharmacother..

[16] Ito T, Shimada Y, Kan T, David S, Cheng Y, Mori Y (2008). Pituitary tumor-transforming 1 increases cell motility and promotes lymph node metastasis in esophageal squamous cell carcinoma. Cancer Res.

[17] Wang F, Liu Y, Chen Y (2016). Pituitary tumor transforming gene-1 in non-small cell lung cancer: Clinicopathological and immunohistochemical analysis. Biomed Pharmacother.

[18] Tsherniak A, Vazquez F, Montgomery PG, Weir BA, Kryukov G, Cowley GS (2017). Defining a Cancer Dependency Map. Cell..

[19] Vazquez F, Sellers WR (2021). Are CRISPR screens providing the next generation of therapeutic targets?. Cancer Res.

[20] Li J, Yuan S, Norgard RJ, Yan F, Sun YH, Kim IK (2021). Epigenetic and transcriptional control of the epidermal growth factor receptor regulates the tumor immune microenvironment in pancreatic cancer. Cancer Disc.

[21] Becht E, Giraldo NA, Lacroix L, Buttard B, Elarouci N, Petitprez F (2016). Estimating the population abundance of tissue-infiltrating immune and stromal cell populations using gene expression. Genome Biol.

[22] Geistlinger L, Oh S, Ramos M, Schiffer L, LaRue RS, Henzler CM (2020). Multiomic analysis of subtype evolution and heterogeneity in high-grade serous ovarian carcinoma. Cancer Res.

[23] Lai H, Cheng X, Liu Q, Luo W, Liu M, Zhang M (2021). Single-cell RNA sequencing reveals the epithelial cell heterogeneity and invasive subpopulation in human bladder cancer. Int J Cancer.

[24] Davis CA, Hitz BC, Sloan CA, Chan ET, Davidson JM, Gabdank I (2018). The Encyclopedia of DNA elements (ENCODE): data portal update. Nucleic Acids Res.

[25] Zheng R, Wan C, Mei S, Qin Q, Wu Q, Sun H (2019). Cistrome Data Browser: expanded datasets and new tools for gene regulatory analysis. Nucleic Acids Res.

[26] Szklarczyk D, Gable AL, Lyon D, Junge A, Wyder S, Huerta-Cepas J (2019). STRING v11: protein-protein association networks with increased coverage, supporting functional discovery in genome-wide experimental datasets. Nucleic Acids Res.

[27] Modhukur V, Iljasenko T, Metsalu T, Lokk K, Laisk-Podar T, Vilo J (2018). MethSurv: a web tool to perform multivariable survival analysis using DNA methylation data. Epigenomics..

[28] Anuraga G, Wang WJ, Phan NN, An Ton NT, Ta HDK, Berenice Prayugo F (2021). Potential prognostic biomarkers of NIMA (Never in Mitosis, Gene A)-Related Kinase (NEK) family members in breast cancer. J Personalized Med.

[29] Zheng Q, Wang Z, Zhang M, Yu Y, Chen R, Lu T (2021). Prognostic value of SEC61G in lung adenocarcinoma: a comprehensive study based on bioinformatics and in vitro validation. BMC Cancer.

[30] Subramanian A, Narayan R, Corsello SM, Peck DD, Natoli TE, Lu X (2017). A next generation connectivity map: l1000 platform and the first 1,000,000 profiles. Cell..

[31] Shah U, Patel A, Patel S, Patel M, Patel A, Patel S (2022). Role of natural and synthetic flavonoids as potential aromatase inhibitors in breast cancer: structure-activity relationship perspective. Anti Cancer Agents Med Chem.

[32] Kharb R, Haider K, Neha K, Yar MS (2020). Aromatase inhibitors: Role in postmenopausal breast cancer. Arch Pharm.

[33] Mallery SR, Wang D, Santiago B, Pei P, Bissonnette C, Jayawardena JA (2019). Fenretinide, tocilizumab, and reparixin provide multifaceted disruption of oral squamous cell carcinoma stem cell properties: implications for tertiary chemoprevention. Mol Cancer Ther.

[34] Hernández-Camarero P, López-Ruiz E, Marchal JA, Perán M (2021). Cancer: a mirrored room between tumor bulk and tumor microenvironment. J Exp Clin Cancer Res CR.

[35] Khalaf K, Hana D, Chou JT, Singh C, Mackiewicz A, Kaczmarek M (2021). Aspects of the tumor microenvironment involved in immune resistance and drug resistance. Front Immunol.

[36] Sivori S, Pende D, Quatrini L, Pietra G, Della Chiesa M, Vacca P (2021). NK cells and ILCs in tumor immunotherapy. Mol Asp Med.

[37] Levy D, Ferreira M, Reichert CO, de Almeida LV, Brocardo G, Lage L (2020). Cell cycle changes, DNA ploidy, and PTTG1 gene expression in HTLV-1 patients. Front Microbiol.

[38] Stoika R, Melmed S (2002). Expression and function of pituitary tumour transforming gene for T-lymphocyte activation. Br J Haematol.

[39] Chiriva-Internati M, Mirandola L, Figueroa JA, Yu Y, Grizzi F, Kim M, et al. Selective expression and immunogenicity of the cancer/testis antigens SP17, AKAP4 and PTTG1 in non-small cell lung cancer: new candidates for active immunotherapy. Chest. 2014. 10.1378/chest.13-0770 24811938

[40] Tian X, Xu WH, Xu FJ, Li H, Anwaier A, Wang HK (2022). Identification of prognostic biomarkers in papillary renal cell carcinoma and PTTG1 may serve as a biomarker for predicting immunotherapy response. Ann Med.

[41] Spachmann PJ, Azzolina V, Weber F, Evert M, Eckstein M, Denzinger S (2020). Loss of CHEK2 Predicts Progression in Stage pT1 Non-Muscle-Invasive Bladder Cancer (NMIBC). Pathol Oncol Res.

[42] Słojewski M, Złowocka E, Cybulski C, Górski B, Debniak T, Wokołorczyk D (2008). CHEK2 germline mutations correlate with recurrence rate in patients with superficial bladder cancer. Ann Acad Med Stetin.

[43] Sinha S, Bheemsetty VA, Inamdar MS (2018). A double helical motif in OCIAD2 is essential for its localization, interactions and STAT3 activation. Sci Rep.

[44] Liu Y, Song C, Ni H, Jiao W, Gan W, Dong X (2018). UBE2L3, a susceptibility gene that plays oncogenic role in hepatitis B-related hepatocellular carcinoma. J Viral Hepat.

[45] Cui Z, Sun S, Li J, Li J, Sha T, He J, et al. UBE2L3 promotes squamous cell carcinoma progression in the oral cavity and hypopharynx via activating the NF-κB signaling by increasing IκBα degradation. Cell Biol Int. 2022. 10.1002/cbin.11772 35128752

[46] Tao NN, Zhang ZZ, Ren JH, Zhang J, Zhou YJ, Wai Wong VK (2020). Overexpression of ubiquitin-conjugating enzyme E2 L3 in hepatocellular carcinoma potentiates apoptosis evasion by inhibiting the GSK3β/p65 pathway. Cancer Lett.

[47] Zeng H, Li T, Zhai D, Bi J, Kuang X, Lu S (2020). ZNF367-induced transcriptional activation of KIF15 accelerates the progression of breast cancer. Int J Biol Sci.

[48] Tong Y, Eigler T (2009). Transcriptional targets for pituitary tumor-transforming gene-1. J Mol Endocrinol.

[49] Pei L (2001). Identification of c-myc as a down-stream target for pituitary tumor-transforming gene. J Biol Chem.

[50] Lin X, Yang Y, Guo Y, Liu H, Jiang J, Zheng F (2019). PTTG1 is involved in TNF-α-related hepatocellular carcinoma via the induction of c-myc. Cancer Med.

[51] Bernal JA, Luna R, Espina A, Lázaro I, Ramos-Morales F, Romero F (2002). Human securin interacts with p53 and modulates p53-mediated transcriptional activity and apoptosis. Nat Genet.

[52] Tong Y, Tan Y, Zhou C, Melmed S (2007). Pituitary tumor transforming gene interacts with Sp1 to modulate G1/S cell phase transition. Oncogene..

