# Supplementary material for: Clinical significance and potential regulatory mechanism of overexpression of pituitary tumor-transforming gene transcription factor in bladder cancer
Source: BMC Cancer. 2022 Jun 29;22:713. doi: 10.1186/s12885-022-09810-y (PMC9241226; doi:10.1186/s12885-022-09810-y)
Supplement: Supplementary file 1 — Additional file 1. [file 12885_2022_9810_MOESM1_ESM.docx]

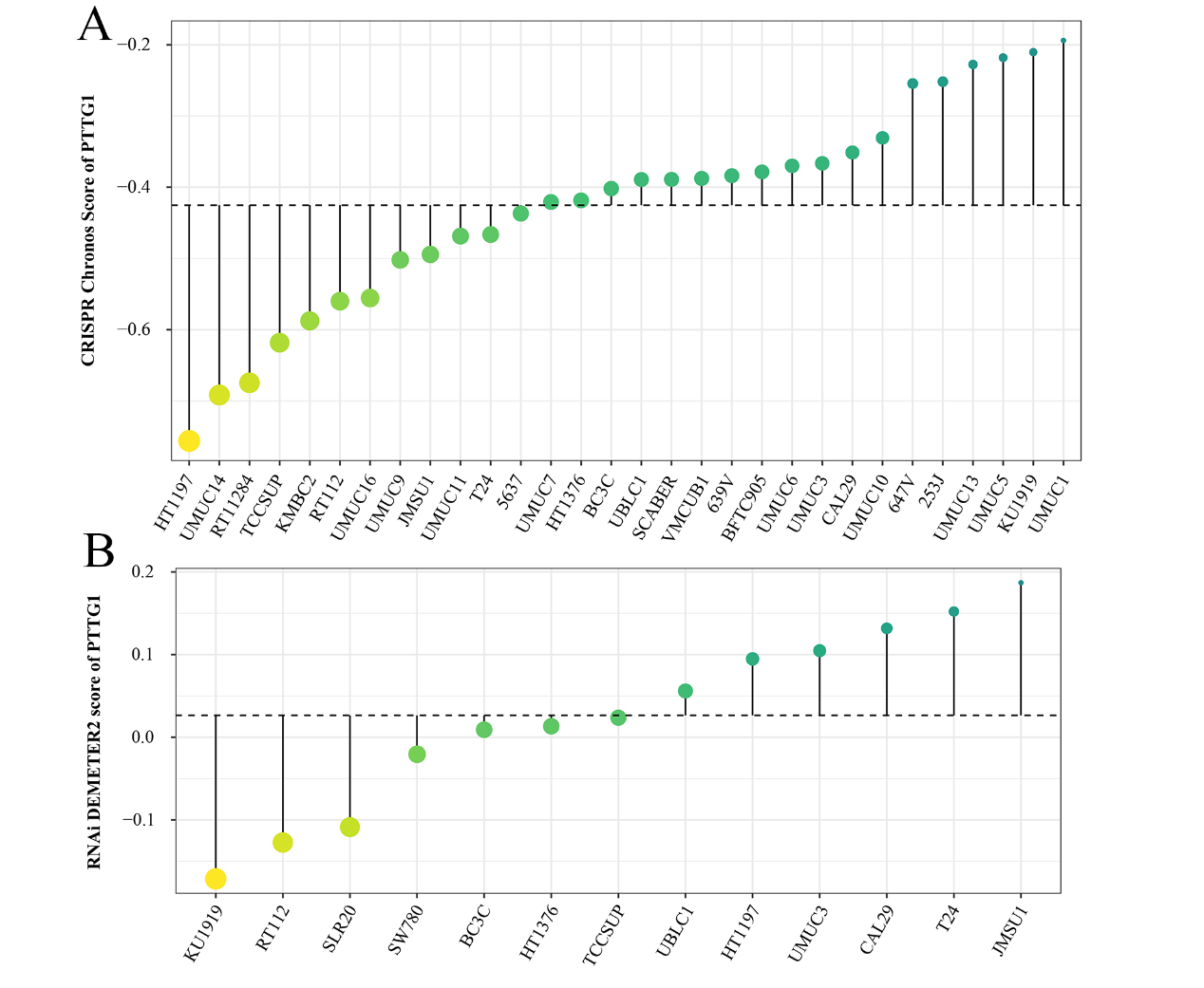


**Figure S1.** Functional verification of PTTG1 as an oncogene in BLCA cell lines

A. Clustered regularly interspaced short palindromic repeats (CRISPR) screen analysis. B. RNA interference (RNAi) screen analysis. The CRISPR Chronos and RNAi DEMETER2 scores are based on the result of the cell deletion assay. CRISPR and RNAi knockout showed that PTTG1 was essential for the survival of BLCA cell lines. BLCA, bladder carcinoma.


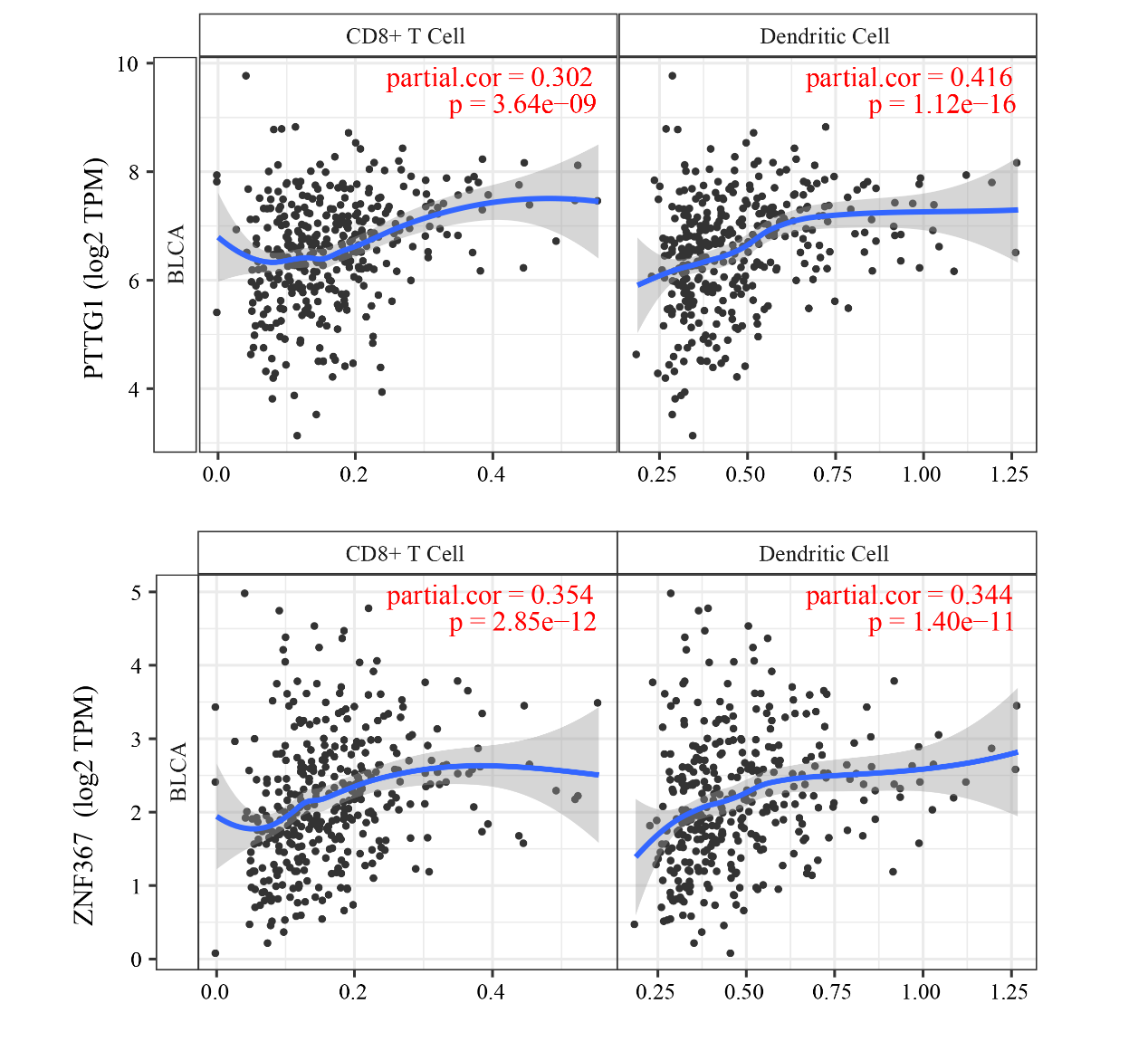


**Figure S2.** A positive association between PTTG1 and immune cell infiltration in BLCA tissues

The TIMER analysis tool was used to verify the correlation between PTTG1 targeting genes with immune cells. Both PTTG1 and ZNF367 were positively correlated to CD8^+^ T cells and dendritic cells. BLCA, bladder carcinoma.


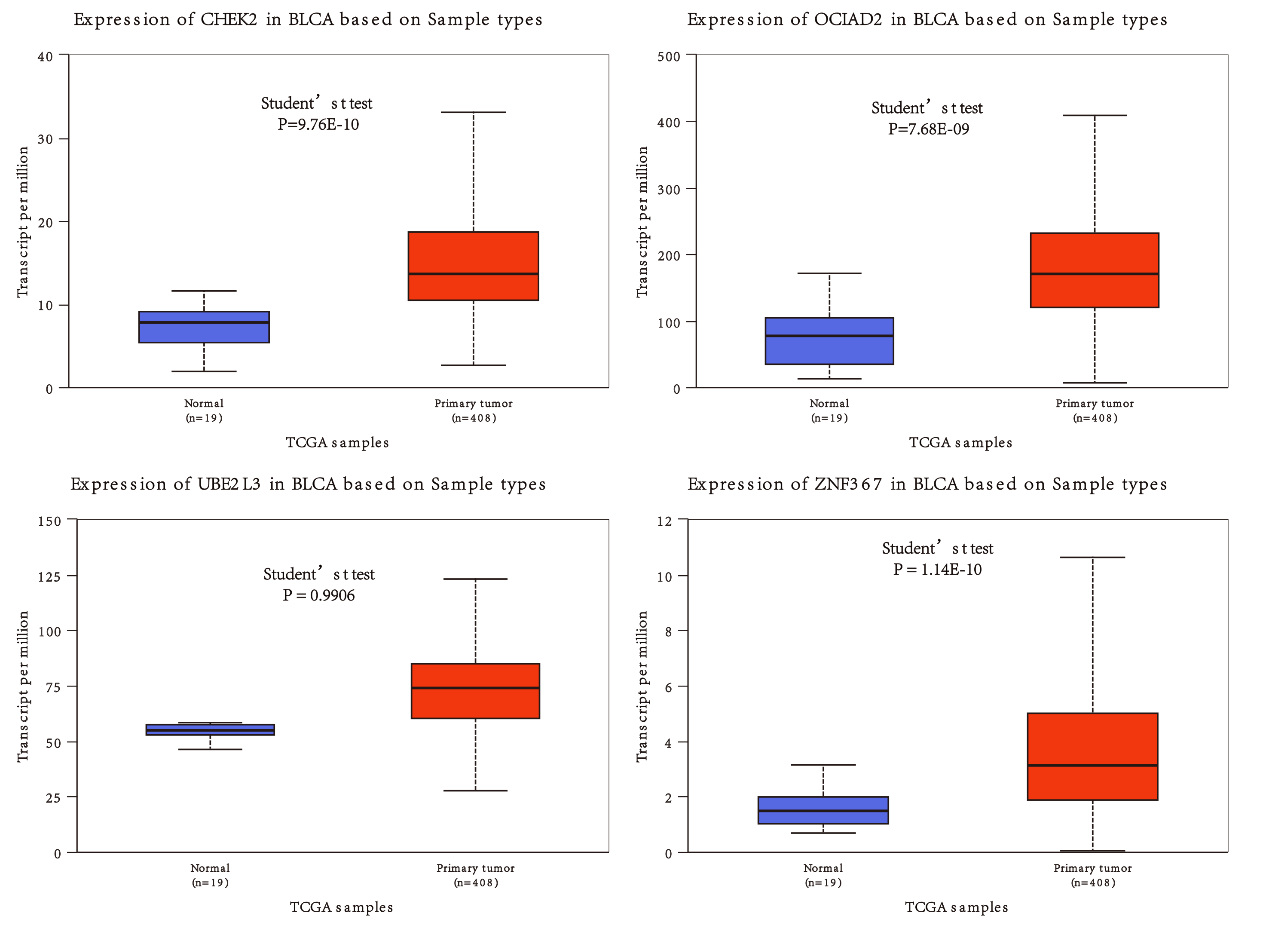


**Figure S3.** Increased expression levels of PTTG1 transcriptional targets in the TCGA-BLCA cohort.

*CHEK2*, *OCIAD2*, *UBE2L3*, and *ZNF367* were upregulated in BLCA tissues. BLCA, bladder carcinoma.


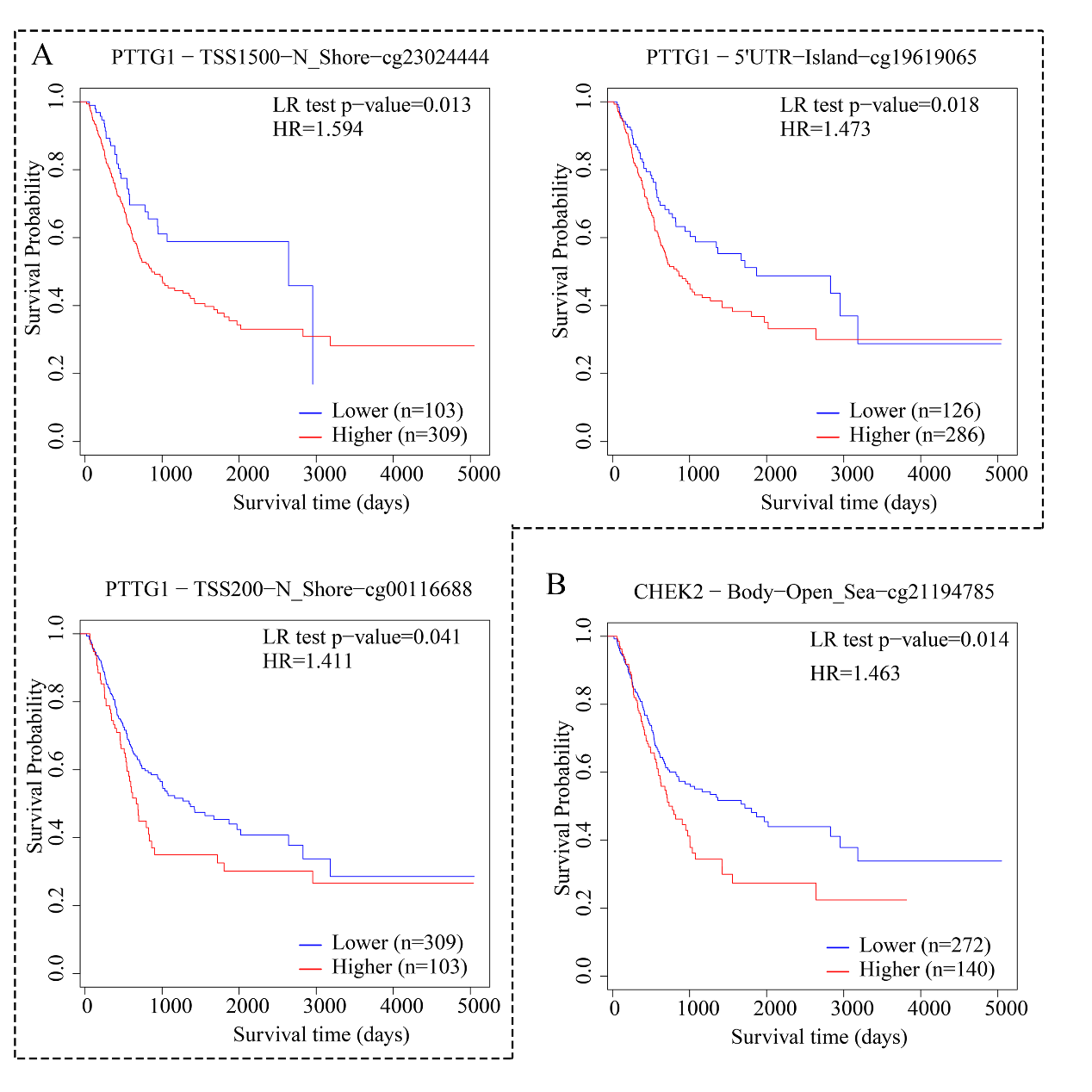


**Figure S4.** Prognostic value of PTTG1 methylation in TCGA-BLCA cohort

The prognostic value of PTTG1, as well as its transcriptional targets, were evaluated based on TCGA-BLCA methylation data. A. A higher methylation level of PTTG1 presaged a poorer prognosis in BLCA patients. B. CHEK2 was predicted as a transcriptional target of PTTG1. A higher methylation level of CHEK2 presaged a poorer prognosis in BLCA patients. BLCA, bladder carcinoma.


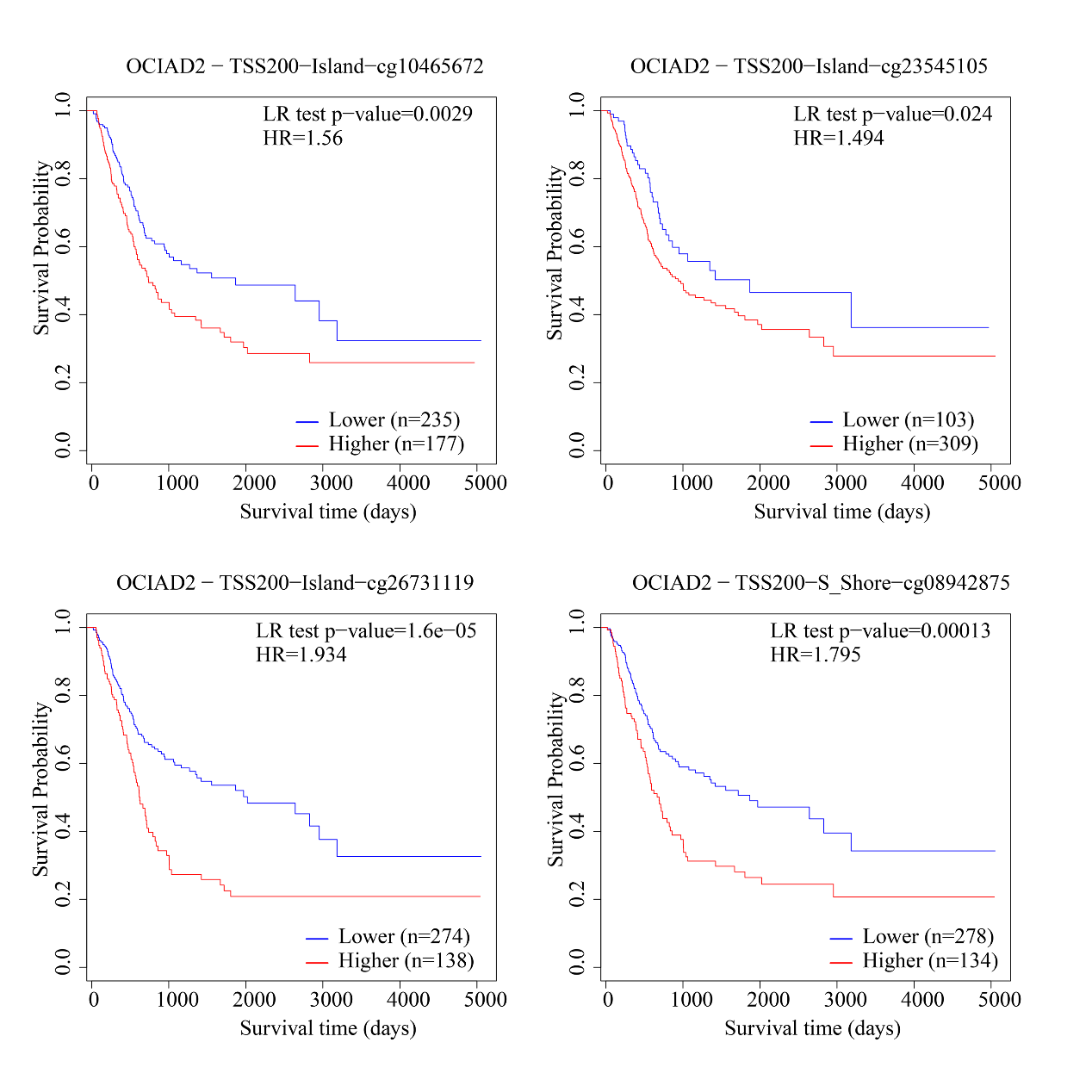


**Figure S5.** Prognostic value of PTTG1 methylation in TCGA-BLCA cohort.

The prognostic value of PTTG1, as well as its transcriptional targets, were evaluated based on TCGA-BLCA methylation data. OCIAD2 was predicted as a transcriptional target of PTTG1. A higher methylation level of OCIAD2 presaged a poorer prognosis in BLCA patients. BLCA, bladder carcinoma.


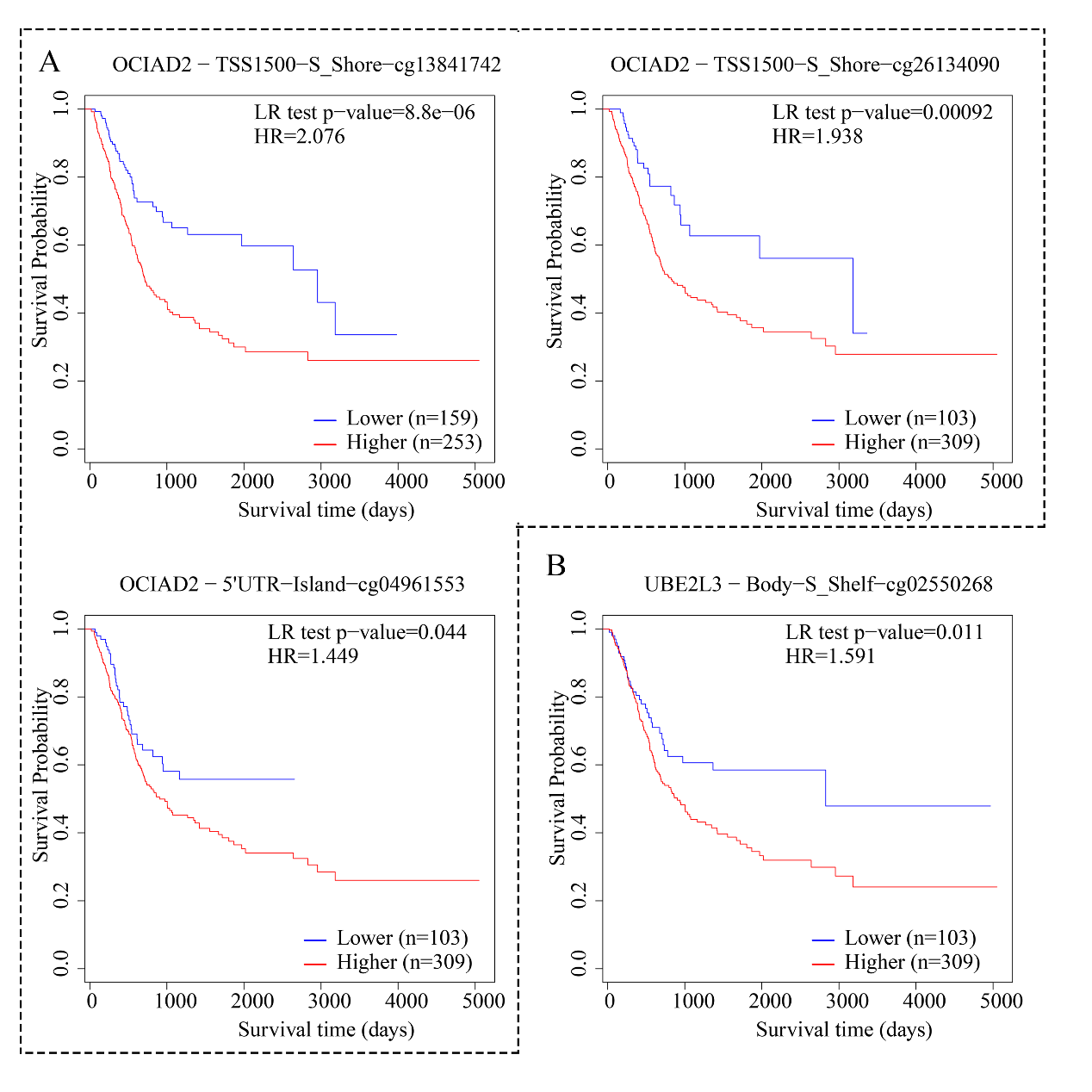


**Figure S6.** Prognostic value of PTTG1 methylation in TCGA-BLCA cohort.

The prognostic value of PTTG1, as well as its transcriptional targets, were evaluated based on TCGA-BLCA methylation data. Both OCIAD2 and UBE2L3 were predicted as transcriptional targets of PTTG1. A. A higher methylation level of OCIAD2 presaged a poorer prognosis in BLCA patients. B. A higher methylation level of UBE2L3 presaged a poorer prognosis in BLCA patients. BLCA, bladder carcinoma.


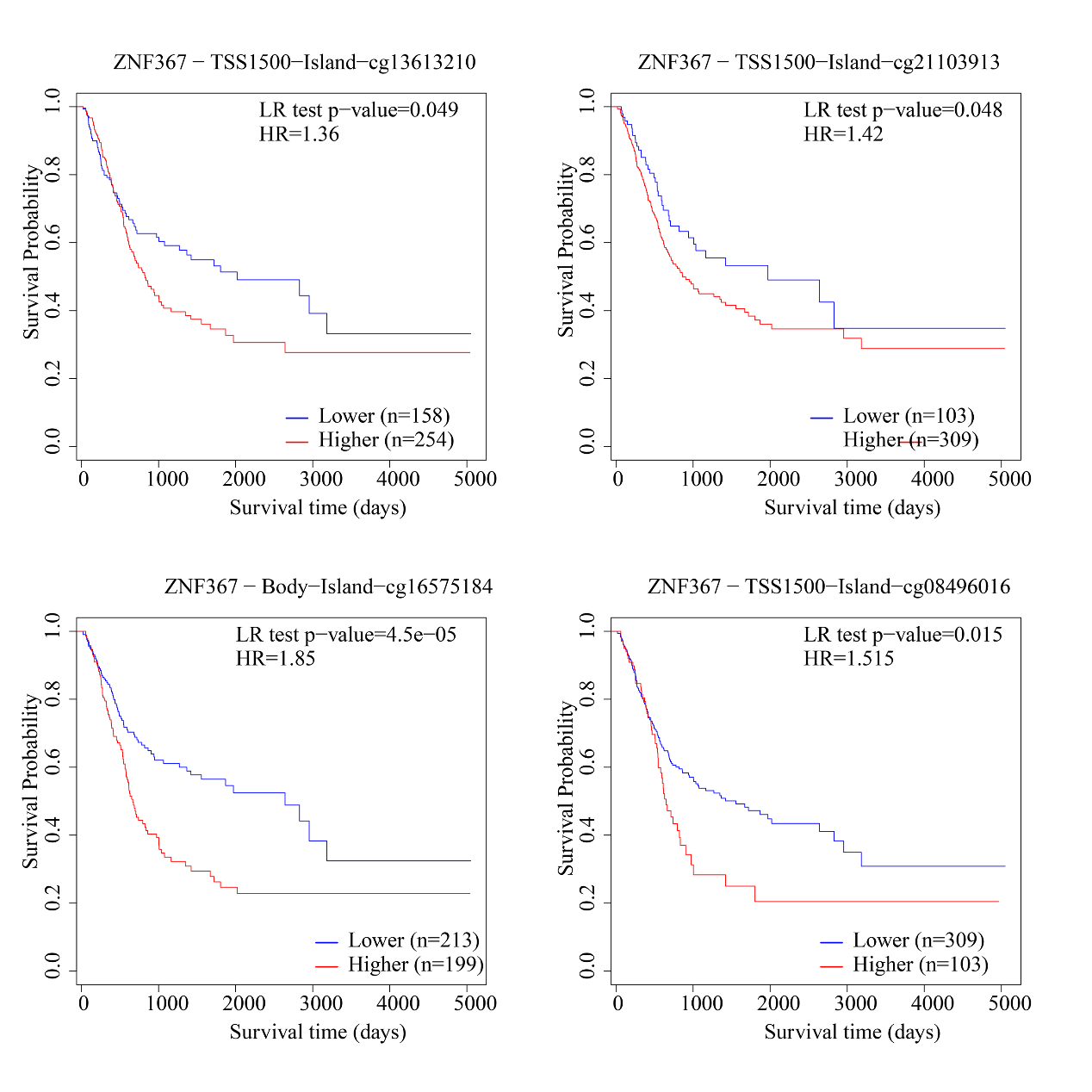


**Figure S7.** Prognostic value of PTTG1 methylation in TCGA-BLCA cohort.

The prognostic value of PTTG1, as well as its transcriptional targets, were evaluated based on TCGA-BLCA methylation data. ZNF367 was predicted as a transcriptional target of PTTG1. A higher methylation level of ZNF367 presaged a poorer prognosis in BLCA patients. BLCA, bladder carcinoma.
